# Seeing the unseen: spatio-temporal visualization of reactive carbocation intermediates in electrolytic cells

**DOI:** 10.1039/d5sc06447c

**Published:** 2025-10-06

**Authors:** Abhijit Nandy, Barsha Pathak, Bikash Ranjan Isaac, Vijayamohanan Pillai, Shibdas Banerjee

**Affiliations:** a Department of Chemistry, Indian Institute of Science Education and Research Tirupati Tirupati 517619 India shibdas@iisertirupati.ac.in vijay@iisertirupati.ac.in

## Abstract

In an electrolytic cell, reactive carbocations often emerge as key intermediates during oxidative electrochemical transformations, particularly near the anode. Owing to their short lifetimes and high electrophilicity, these species are typically challenging to detect directly *in situ*. This study employed spatial and temporal sampling of some electrochemically driven C–O, C–N, and C–C bond-forming reactions, followed by rapid analysis of the collected aliquots using desorption electrospray ionization mass spectrometry. The captured carbocations were then visualized through a contour plot representing their abundance across the electrolytic cell. This spatio-temporal resolution of reactive intermediates enables transforming electrochemical studies from static end-point analysis to dynamic, mechanistically rich investigations, offering detailed insights into the formation, transformation, and diffusion of key intermediates in spatial and temporal dimensions.

## Abstract 2

Reactive carbocations generated in the electrolytic cell were visualized *via* mass spectrometry imaging, enabling spatiotemporal mapping of the reaction zone and providing mechanistic insights into electroorganic synthesis.

## Graphical abstract

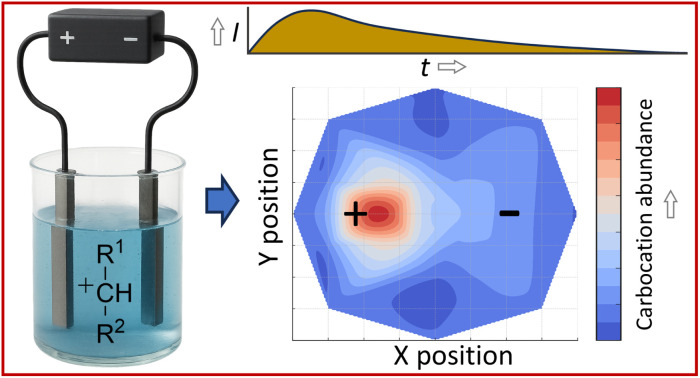

## Introduction

Electroorganic chemistry has gained significant attention as a sustainable and versatile approach for driving organic transformations using electricity.^1–10^ While many mechanistic interpretations in electroorganic synthesis remain speculative, leading to a substantial gap between proposal and experimental validation, the ability to directly detect and characterize the fleeting electrochemical intermediates is crucial for bridging this gap. Cyclic voltammetry (CV), Fourier-transform infrared spectroscopy, and surface-enhanced Raman spectroscopy often provide valuable information about the electrochemical reaction mechanism.^11–23^ However, signal averaging, interference from side reactions, overlapping redox peaks, and the insensitivity of CV to short-lived or non-electroactive intermediates highlight the need for complementary techniques capable of real-time monitoring of reactive intermediates in electrochemical reactions (Note S1). Although, in recent years, a variety of electrochemically generated reactive species, such as radical ions, nitrenium ions, neutral species, and transient metal complexes, have been probed by mass spectrometry,^24–35^ the direct detection of highly reactive carbocation intermediates from an electrochemical reaction vessel remains largely unexplored.^36^ The present investigation offers unprecedented insights into the real-time behavior, stability, and dynamic movement of reactive carbocation intermediates in an electrolytic environment, advancing our understanding of the spatial characteristics of reactions occurring inside the electrolytic cell.

Recently, our group demonstrated that electrohydrodynamically generated water microdroplets are super acidic and can directly capture and stabilize a wide range of reactive carbocation intermediates, which are otherwise highly unstable in associated bulk reaction media.^37–43^ This was followed by their direct interception and detection from the reaction medium using mass spectrometry, enabling real-time evaluation of the corresponding chemical transformations. While all those earlier studies involving desorption electrospray ionization mass spectrometry (DESI-MS) were limited to investigating the reactive intermediates in the conventional reaction vials, the present work seeks to extend our approach to an electrolytic cell, with the goal of profiling reactive carbocation and radical cation intermediates across both spatial and temporal dimensions. When integrated with temporal analysis, spatial profiling allows a spatio-temporal resolution of the reactive intermediates, which is critical for understanding the dynamic behavior of the redox transformations not only in the vicinity of the electrode but throughout the entire reaction chamber.

## Results and discussion

**Fig. 1:**
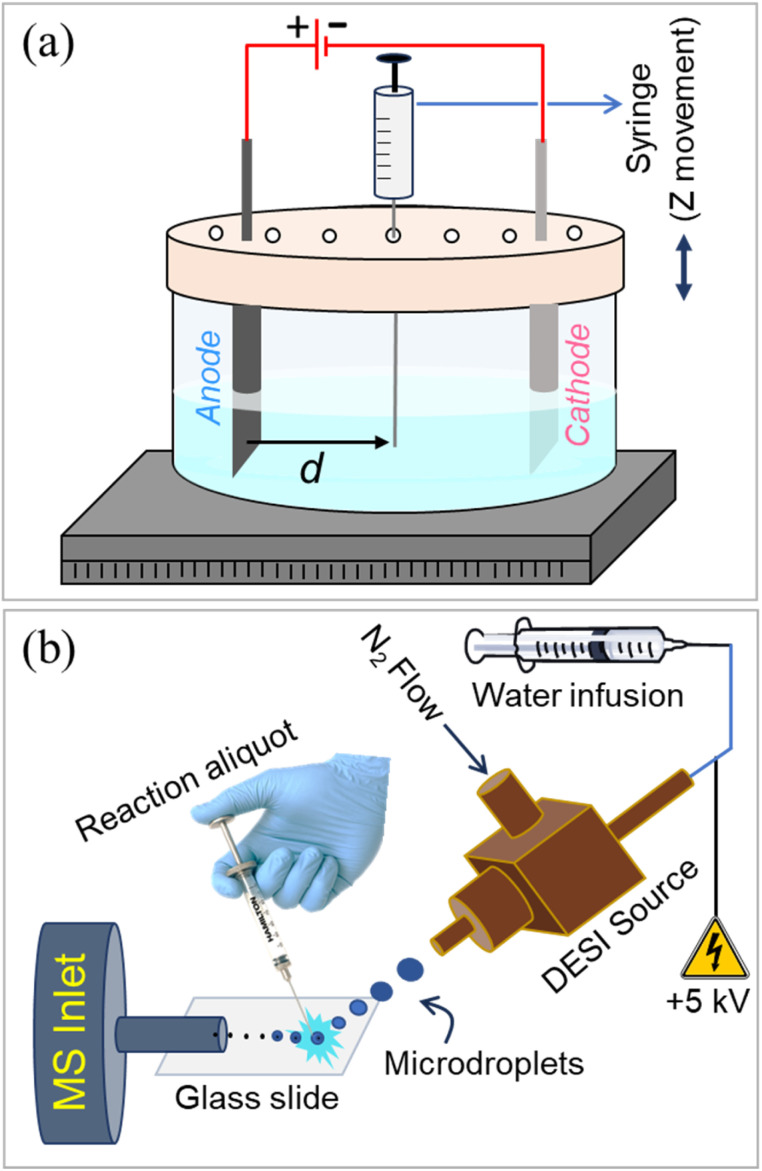
(a) Diagram of the electrochemical reaction chamber with pre-defined sampling ports (holes) on the lid, strategically located at different distances from the anode (*d*) to facilitate spatial sampling during the reaction. (b) A 10 μL reaction aliquot withdrawn from the electrolytic cell was subjected to bombardment with positively charged water microdroplets in a DESI-MS setup, enabling rapid interception and detection of the reactive carbocation intermediate.

**Fig. 2:**
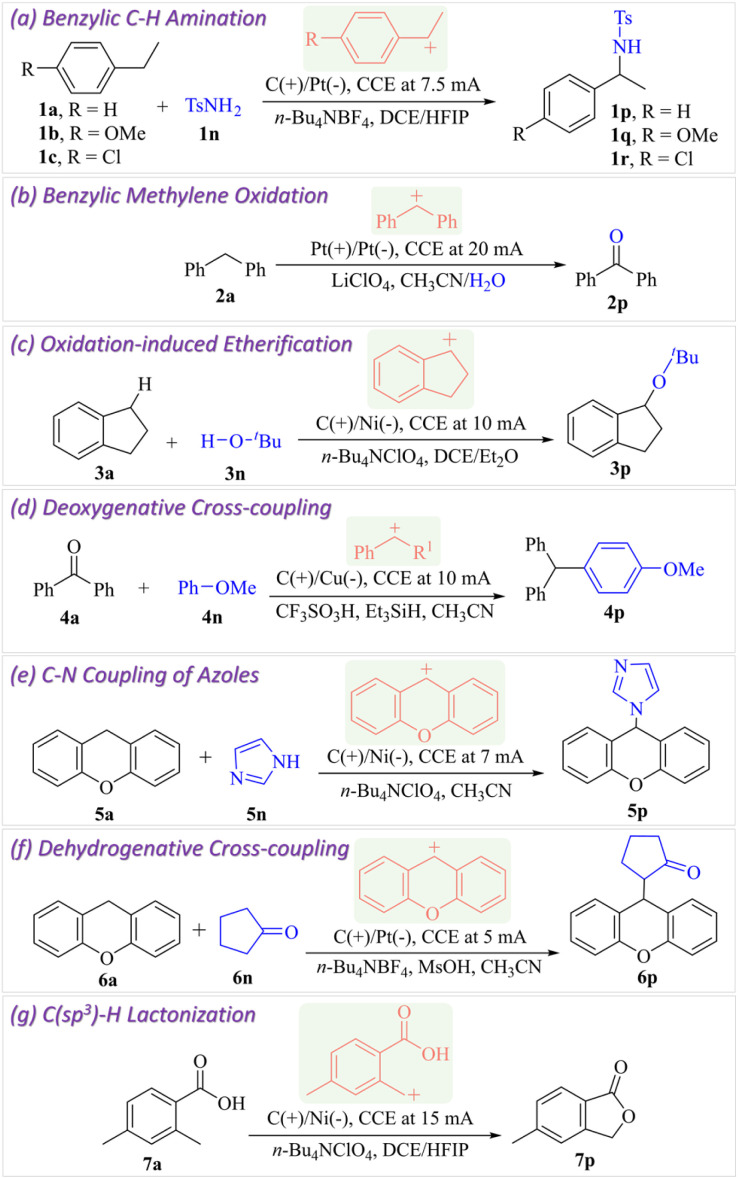
Model electrochemical reactions carried out to generate different types of transient carbocation intermediates (highlighted in red). CCE refers to constant current electrolysis.

Fig. 1a illustrates the experimental setup featuring a custom-designed electrochemical reaction vessel, enabling the withdrawal of 10 μL of the reaction solution by vertically inserting a Hamilton syringe needle through the pre-defined sampling port at various reaction times and spatial locations (Fig. S1). The aliquoted reaction solution was rapidly dispensed on a microscope glass slide, which was kept under an impinging spray of charged water microdroplets in a custom-built DESI-MS source (Fig. 1b). Thus, the reactive species were extracted into the splashed microdroplets and pneumatically propelled toward the mass spectrometer, facilitating their detection. All necessary parameters, including the DESI spray source geometry and associated mass spectrometric conditions, were optimized in our earlier studies and the same conditions were employed in this work.^37,39–41^ We performed six model electrochemical reactions of different types, following established protocols reported in the literature (Fig. 2). The reactions include site-selective benzylic C–H amination,^44^ benzylic methylene oxidation,^45^ oxidation-induced etherification,^46^ deoxygenative cross-coupling,^47,48^ C–N coupling of azoles,^49^ and lactonization.^50^ Based on substrate and product studies, the formation of carbocations was proposed earlier as the key intermediate in the mechanistic pathways of these reactions.

**Fig. 3:**
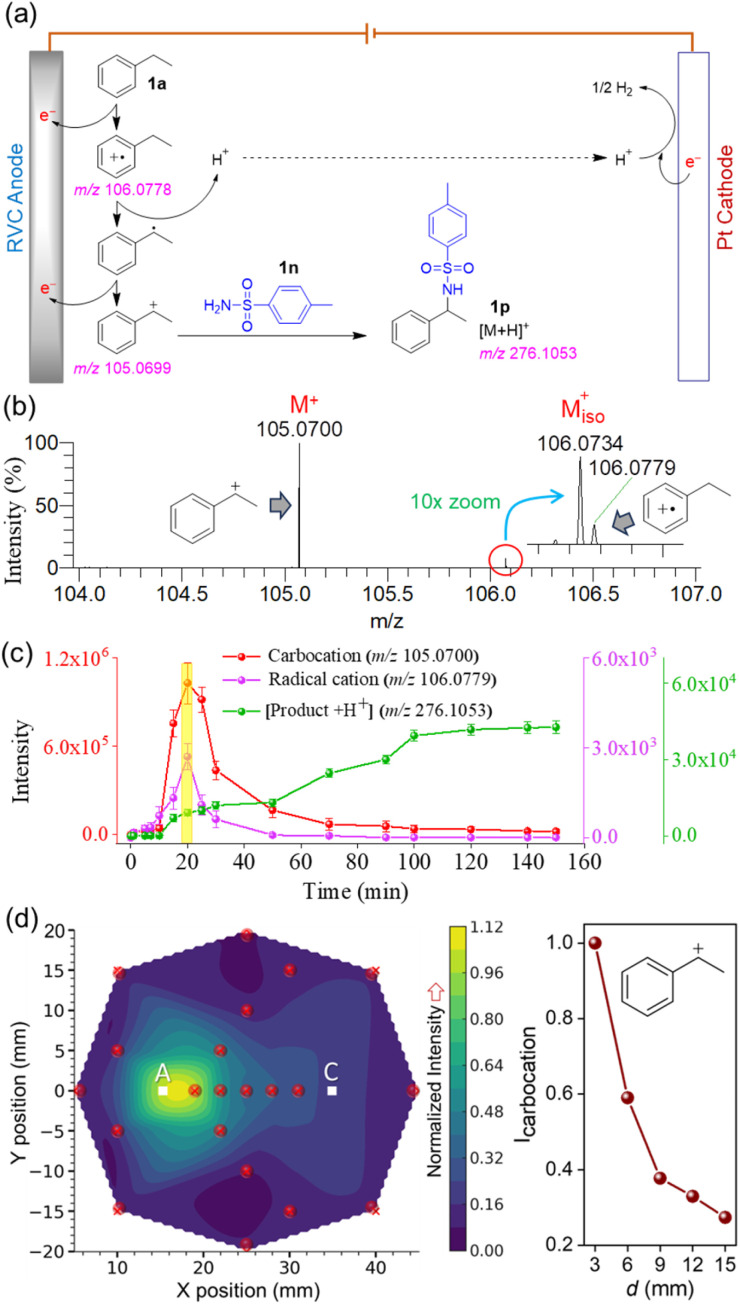
(a) Schematic presentation of carbocation intermediacy in the site-selective electrochemical benzylic C–H activation of ethylbenzene (Fig. 2a) with a plausible mechanism.^44^ (b) DESI-MS detection of the benzylic carbocation intermediate involved in the reaction. (c) Temporal evolution of the sequential formation of the intermediate radical cation, carbocation, and the product in the electrochemical reaction, enabling their real-time monitoring. Data were averaged over three independent replicates. (d) Interpolated contour plot (left panel) showing the spatial distribution of carbocation abundance (normalized to 1) within the electrochemical reaction vessel, measured at the depth aligned with the center of the anode. “A” denotes the anode position and “C” the cathode. The red dot in the contour plot marks the specific sampling point. Right panel shows the decrease in carbocation intensity as the sampling location moves from the anode to the cathode.

We began our investigation with the benzylic C–H amination involving ethylbenzene and *para*-toluenesulfonamide (Fig. 3a). A reticulated vitreous carbon (RVC) electrode served as the anode, while a platinum (Pt) electrode functioned as the cathode. The reaction was conducted under a constant current of 7.5 mA. The substrate (1a) oxidation near the anode forms a radical cation species, which is converted to a benzylic carbocation intermediate, which is subsequently captured by the nucleophile (1n) to yield the C–H/N–H cross-coupling product 1p (Fig. 3a).^44^ As the reaction progressed, a small volume (10 μL) of the reaction aliquot was screened in the DESI source (Fig. 1), enabling the real-time detection of both intermediate species in the corresponding mass spectrum (Fig. 3b). The high mass-to-charge (*m*/*z*) accuracy (Table S1) and the resolution of the mass spectrometer facilitated the reliable detection of these fleeting intermediates. Detailed insights into the stability of carbocations at the air–water interface of microdroplets in the DESI source and their subsequent transfer to the gas phase for mass spectrometric detection can be found in our earlier reports.^37,39–41^ Thus, we intercepted the above two reactive cationic species at various time points during the reaction and evaluated their temporal abundance at a position 3 mm from the anode (toward the cathode side), at a depth aligned with the center of the anode.

This temporal profiling (Fig. 3c) revealed that the abundance of the carbocation intermediates initially increased as the reaction proceeded, peaked at around 20 minutes, and then gradually declined over time (red line). A comparable trend was observed for the precursor radical cation species (pink line). The concomitant formation of the product exhibited a steady increase over time, eventually reaching saturation at approximately 100 minutes (green line), indicating nearly completion of the reaction. This reaction profile contrasts sharply with the control study, where all reactants were mixed without applying any current, and no ion signals corresponding to the carbocation, radical cation, or product were detected (Fig. S2). These results therefore rule out the possibility that the observed species (intermediates and products) were generated in the DESI source by microdroplet-induced chemistry.^51–55^ These findings unequivocally confirm the involvement of the benzylic carbocation as a key intermediate in the electrochemical reaction. To investigate the spatial distribution of this reactive intermediate across the electrolytic cell, we planned to generate a mass spectrometry image visualizing its spatial abundance. We sampled the reaction aliquot from several pre-defined locations with respect to the anode and at a depth aligned with the center of the anode (see Materials and methods for details). Accordingly, 21 aliquots of 10 μL each were sequentially sampled and analyzed using DESI-MS (Fig. 1) within a 3.5 minute window, corresponding to the period when the reaction exhibited the high abundance of the benzylic cation species (18 to 21.5 minutes, as highlighted in yellow in Fig. 3c). Fig. 3d presents the interpolated contour plot of carbocation signal intensity, depicting its spatial distribution throughout the cylindrical electrolytic cell. This ion image reveals that carbocation abundance is highest in the vicinity of the anode and progressively diminishes toward the periphery of the reaction vessel. Furthermore, the right panel of Fig. 3d shows a sharp decline in carbocation intensity as the sampling location moves from the anode toward the cathode. Notably, the reactive carbocation signal remains detectable even at the farthest sampling point from the anode, suggesting that carbocations, though primarily generated near the anode, can diffuse across various regions of the reaction vessel before their annihilation (*e.g.*, reaction with the nucleophile). This also reflects the notable stability of the carbocation in the electrolyte-rich reaction medium, allowing it to persist through the aliquoting process until detection. While partial annihilation of the carbocation within the Hamilton syringe during sampling is possible, any such loss is expected to be proportionally consistent across all spatial and temporal sampling points, and is therefore unlikely to impact the overall findings of this study. In another set of experiments, carbocation detection from this electrochemical reaction (Fig. 3a) was assessed by changing the droplet pH, solvent, and spray voltage in the DESI source (Fig. S3). The results suggest that the superacidic water microdroplet surface is critical for the efficient desorption and stabilization of carbocations, aligning well with our previous findings.^37–40^ When we carried out analogous benzylic C–H amination reactions using two different substrates (1b and 1c in Fig. 2a), the respective reactive carbocation intermediates were consistently captured under comparable experimental conditions (Fig. S4 and S5), with both temporal and spatial resolution (from the anode to the cathode).

**Fig. 4:**
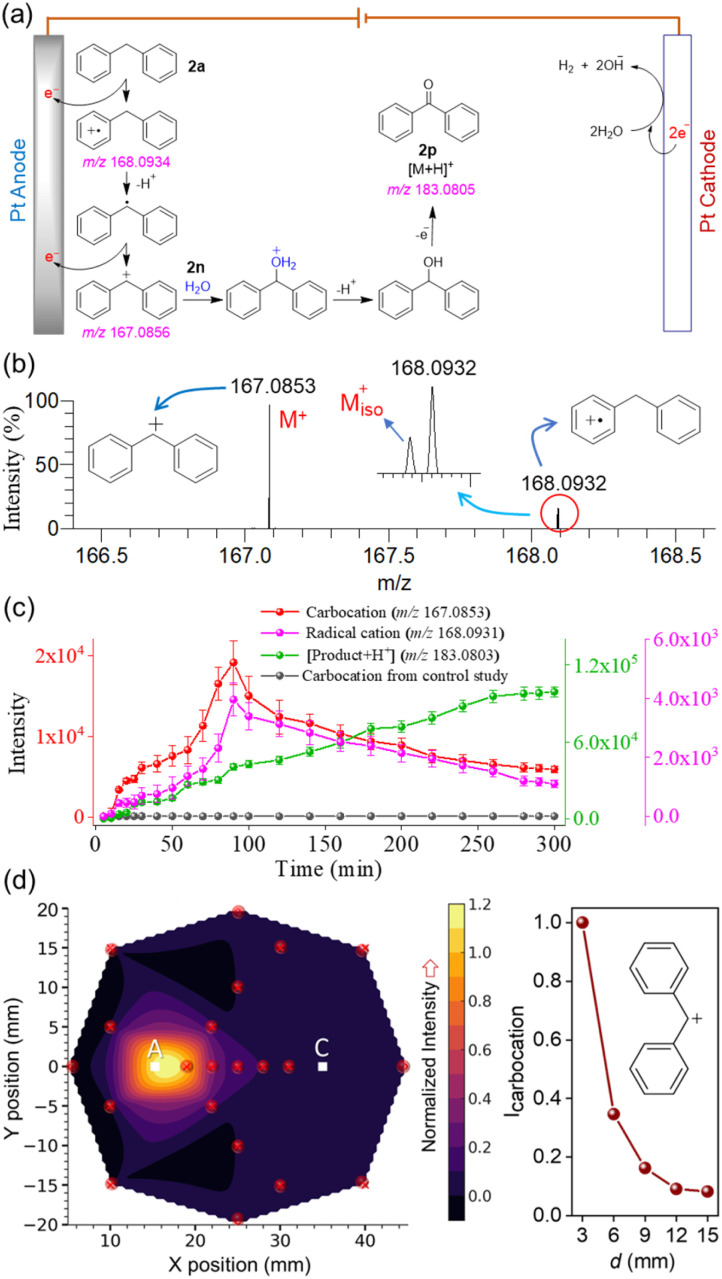
(a) Schematic presentation of carbocation intermediacy in the electrooxidative C–H activation of diphenylmethane (Fig. 2b) with a plausible mechanism.^45^ (b) DESI-MS detection of the diphenylmethylium carbocation intermediate involved in the reaction. (c) Temporal evolution of the sequential formation of the intermediate radical cation, carbocation, and the product in the electrochemical reaction, enabling their real-time monitoring. Data were averaged over three independent replicates. (d) Interpolated contour plot (left panel) showing the spatial distribution of carbocation abundance (normalized to 1) within the electrochemical reaction vessel, measured at the depth aligned with the center of the anode. “A” denotes the anode position and “C” the cathode. The red dot in the contour plot marks the specific sampling point. Right panel shows the decrease in carbocation intensity as the sampling location moves from the anode to the cathode.

Likewise, we investigated the electrooxidative C–H activation of diphenylmethane (Fig. 2a), which led to ketone formation *via* radical cation and carbocation intermediates (Fig. 4a).^45^Fig. 4b shows the ion signals corresponding to the carbocation and radical cation species captured from the electrolytic cell as reactive intermediates. The high mass accuracies of these species are listed in Table S1.

The abundances of the intermediate radical cation, carbocation, and the final product are plotted as a function of reaction time (Fig. 4c). This temporal profiling revealed a rise in carbocation intensity up to 90 minutes, followed by a gradual decline as the reaction proceeded (red trace, Fig. 4c). Concurrent monitoring of the radical cation and the reaction product facilitated detailed tracking of the reaction course, providing real-time kinetic and mechanistic insights. Although a control study (reaction mixture without applied current) detected a trace level signal of the carbocation (Fig. S6), which remained unchanged over time (black line, Fig. 4c), the rise-and-fall abundance of the diphenylmethyl carbocation (Fig. 4c) during electrooxidation delineated its role as a reactive intermediate. Nevertheless, the observed carbocation signal in the control study may be attributed to the weak in-droplet dissociation of the substrate into the reactive carbocation.^42,56^ The spatial profiling of the carbocation across the electrolytic cell is presented as a contour plot in Fig. 4d, which further confirms that the carbocation abundance is highest near the anode and gradually decreases toward the cathode (right panel, Fig. 4d) and the periphery of the reaction chamber.

**Fig. 5:**
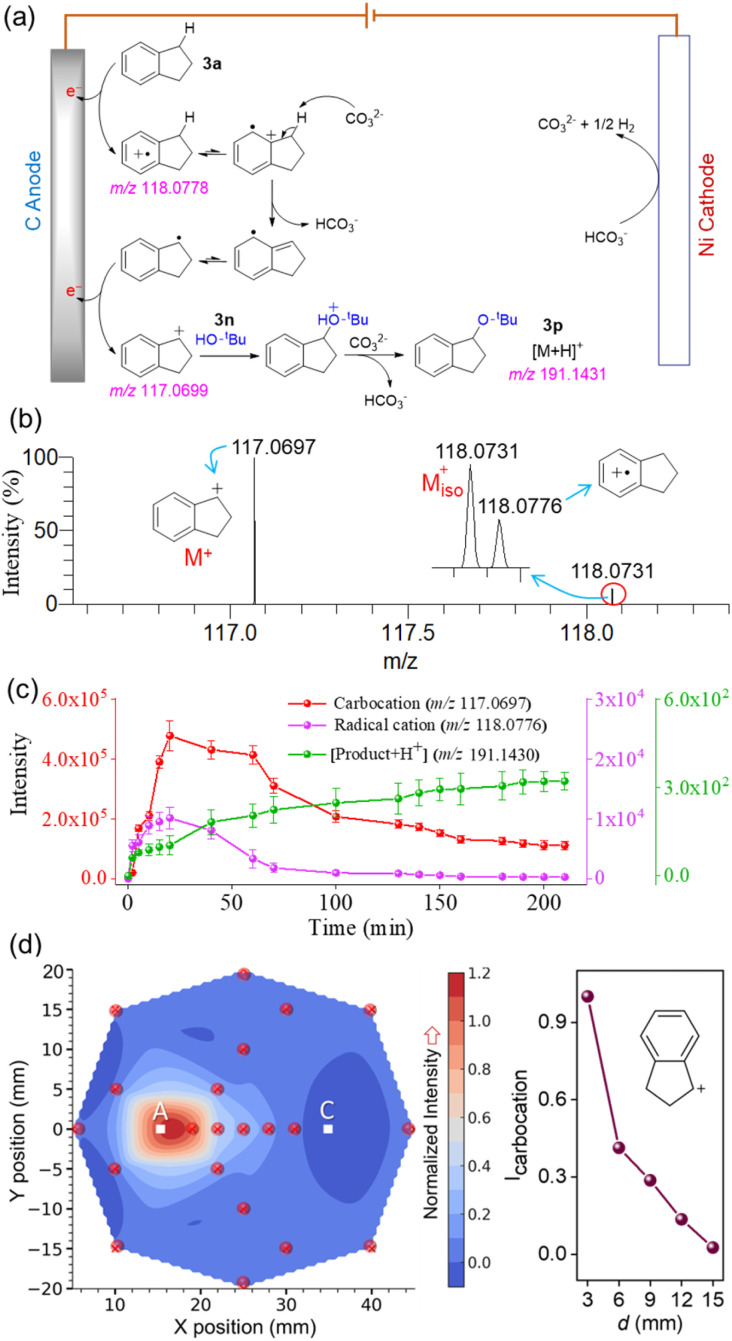
(a) Proposed mechanism involving carbocation intermediacy in site-selective electrochemical etherification of indane (Fig. 2c).^46^ (b) DESI-MS detection of the carbocation intermediate involved in the reaction. (c) Temporal evolution of the sequential formation of the intermediate radical cation, carbocation, and the product in the electrochemical reaction, enabling their real-time monitoring. Data were averaged over three independent replicates. (d) Interpolated contour plot (left panel) showing the spatial distribution of carbocation abundance (normalized to 1) within the electrochemical reaction vessel, measured at the depth aligned with the center of the anode. “A” denotes the anode position and “C” the cathode. The red dot in the contour plot marks the specific sampling point. Right panel shows the decrease in carbocation intensity as the sampling location moves from the anode to the cathode.

Analogous experiments were conducted to detect the carbocation intermediate formed during the electrochemical oxidation-induced etherification of indane (Fig. 2c). The proposed reaction mechanism (Fig. 5a)^46^ was validated by DESI-MS interception of the corresponding radical cation and carbocation intermediates directly from the electrolytic cell (Fig. 5b) with high mass accuracy (Table S1). Temporal abundance profiles of the associated radical cation, carbocation, and product were generated by monitoring their signal intensities at different time intervals (Fig. 5c). The carbocation signal peaked at 20 minutes before declining (red line), with a similar trend observed for the radical cation (pink line), but the product formation steadily increased. In contrast, the control study, conducted without applying current, showed no detectable signals for the carbocation, radical cation, or product (Fig. S7). The spatial abundance profile of the carbocation intermediate, shown as a contour plot (Fig. 5d), reveals higher intensity near the anode, gradually decreasing toward the cathode (right panel, Fig. 5d) and the periphery of the electrolytic cell.

Similar experimental interventions were successfully applied to four additional types of electrochemical reactions (Fig. 2d–g), consistently enabling spatio-temporal resolution of the reactive carbocation intermediates in the electrolytic cell (Fig. S8–S11). These results underscore the robustness and versatility of our approach across diverse reaction systems. Moreover, such electrochemical reaction zone mapping not only offers valuable mechanistic insights through direct detection of intermediates but also facilitates differentiation of sequentially formed species, thereby clarifying the roles of mass transport and diffusion in governing reactivity.

It is important to mention that electrolytic reactions were performed without external stirring in order to preserve the natural spatial and temporal gradients of reactive intermediates across the cell, thereby enabling direct mapping of their localized abundance. Stirring would otherwise homogenize the medium and erase the intrinsic gradients that this study aims to capture. It should also be noted that the total scan time for spatial mapping of the reactive carbocation intermediate was 3.5 min, and therefore the effect of diffusion during this period cannot be excluded. However, given that the overall reaction time ranges from 1 to 5 h (*e.g.*, Fig. 3c, 4c and 5c), and that the mapping was performed within a window where the carbocation abundance was at its maximum, we assumed that the diffusion coefficient of the reactive species does not vary significantly within this short interval,^57^ and thus the observed spatial distribution remains representative and does not compromise the overall conclusions. A critical aspect of the success of this study is that the reactive carbocation survives, at least partially, during the 10 s required for aliquoting prior to DESI-MS scanning. While this observation is not entirely unexpected, since conventional reactions may continue even within the Hamilton syringe after sample withdrawal, as noted in our earlier experiments,^37–40^ the case of the present electrochemical reaction is more thought-provoking and warrants further investigation. To address this, we examined a typical electrochemical transformation (Fig. 2a) using DESI-MS. At a given reaction time, six aliquots of 10 μL each were withdrawn through the same sampling port using separate Hamilton syringes and subsequently analyzed by DESI-MS at six different time points, corresponding to varying residence times of the aliquots in the syringe. As anticipated, the carbocation signal intensity progressively decreased with increasing residence time, reflecting the gradual annihilation of the intermediate in the withdrawn aliquots over a period of approximately 2 min (Fig. S12). Therefore, since the carbocation survives in the Hamilton syringe for a duration longer than the 10 s aliquoting period required for spatial mapping, the results presented here are expected to reliably reflect its spatial distribution (relative) of the intermediate in the electrolytic cell, although a proportionally equal degree of partial annihilation is likely across the 21 aliquots sampled from different ports for spatial mapping (*e.g.*, Fig. 3d, 4d and 5d). Although the reason for the persistence of reactive carbocations in aliquots withdrawn from the electrolytic cell remains uncertain, one plausible explanation is stabilization by the high concentration of supporting electrolyte, particularly in the presence of bulky counter anions (weak nucleophile), which may allow the species to persist in the Hamilton syringe until DESI-MS analysis.

## Conclusions

Carbocations are pivotal intermediates in many transformations in electroorganic synthesis. Traditionally, it has been assumed that such reactive species, owing to their short lifetimes, high reactivity, and localized generation near the electrode, are rapidly quenched by nucleophilic attack in the immediate vicinity of the electrode. In contrast, the present study directly visualizes the reaction profile by spatio-temporal mapping of carbocations across the electrolytic cell. The results reveal that, although the reaction is most intense near the electrode, carbocation-mediated reactivity extends throughout the cell. We further demonstrate that, in the electrolyte-rich environment, these carbocations exhibit a longer persistence than previously recognized, enabling their temporal mapping by sequential aliquoting and subsequent DESI-MS screening. Unlike earlier approaches that rely on static end-point analysis, this strategy provides a dynamic window into real-time mechanistic investigations of electrochemical reactions. By constructing contour plots of carbocation abundance, the method offers a tangible depiction of intermediate lifetimes, spatial distribution, and diffusion behavior in the electrolytic cell. Such visualization not only provides unprecedented mechanistic insights but also lays the foundation for the rational optimization of electroorganic synthesis by directly linking intermediate dynamics with product outcomes. While the present study employed manual sampling, future advancements, such as the integration of automated robotic systems for reaction aliquoting, are expected to enhance precision, enabling faster and more detailed spatial mapping of reactive intermediates across the electrolytic cell. Overall, this development fills a critical gap by providing the first spatio-temporal visualization of carbocations in an electrolytic environment—an achievement not previously attainable with any spectroscopic or electroanalytical method.

## Author contributions

S. B. and V. P. designed the research; A. N., B. P., and B. R. I. performed experiments; A. N., B. P., and S. B. analyzed data; S. B., V. P., and A. N., wrote the paper.

## Conflicts of interest

There are no conflicts to declare.

## Supplementary Material

SC-OLF-D5SC06447C-s001

## Data Availability

The additional data supporting this article are included in the supplementary information (SI). Supplementary information: supplementary methods, experimental setup photographs. See DOI: https://doi.org/10.1039/d5sc06447c.

## References

[cit1] Zhu C., Ang N. W. J., Meyer T. H., Qiu Y., Ackermann L. (2021). Organic Electrochemistry: Molecular Syntheses with Potential. ACS Cent. Sci..

[cit2] Pollok D., Waldvogel S. R. (2020). Electro-organic synthesis – a 21st century technique. Chem. Sci..

[cit3] Rein J., Zacate S. B., Mao K., Lin S. (2023). A tutorial on asymmetric electrocatalysis. Chem. Soc. Rev..

[cit4] Guan W., Chang Y., Lin S. (2023). Electrochemically Driven Deoxygenative Borylation of Alcohols and Carbonyl Compounds. J. Am. Chem. Soc..

[cit5] Yan M., Kawamata Y., Baran P. S. (2017). Synthetic Organic Electrochemical Methods Since 2000: On the Verge of a Renaissance. Chem. Rev..

[cit6] Górski B., Rein J., Norris S., Ji Y., McEuen P. L., Lin S. (2025). Light-harvesting microelectronic devices for wireless electrosynthesis. Nature.

[cit7] Elsherbini M., Wirth T. (2019). Electroorganic Synthesis under Flow Conditions. Acc. Chem. Res..

[cit8] Ali T., Wang H., Iqbal W., Bashir T., Shah R., Hu Y. (2023). Electro-Synthesis of Organic Compounds with Heterogeneous Catalysis. Adv. Sci..

[cit9] Regnier M., Vega C., Ioannou D. I., Noël T. (2024). Enhancing electrochemical reactions in organic synthesis: the impact of flow chemistry. Chem. Soc. Rev..

[cit10] Bu F., Deng Y., Xu J., Yang D., Li Y., Li W., Lei A. (2024). Electrocatalytic reductive deuteration of arenes and heteroarenes. Nature.

[cit11] Mohamadighader N., Zivari-Moshfegh F., Nematollahi D. (2024). Electrochemical generation of phenothiazin-5-ium. A sustainable strategy for the synthesis of new bis(phenylsulfonyl)-10*H*-phenothiazine derivatives. Sci. Rep..

[cit12] Koovakattil Surendran A., Roithová J. (2024). Decoding Voltammograms at the Molecular Frontier: Integration of Voltammetry and Mass Spectrometry. Chem.: Methods.

[cit13] Hoar B. B., Zhang W., Xu S., Deeba R., Costentin C., Gu Q., Liu C. (2022). Electrochemical Mechanistic Analysis from Cyclic Voltammograms Based on Deep Learning. ACS Meas. Sci. Au.

[cit14] Lee C. W., Cho N. H., Nam K. T., Hwang Y. J., Min B. K. (2019). Cyclic two-step electrolysis for stable electrochemical conversion of carbon dioxide to formate. Nat. Commun..

[cit15] Salehi-Khojin A., Jhong H.-R. M., Rosen B. A., Zhu W., Ma S., Kenis P. J. A., Masel R. I. (2013). Nanoparticle Silver Catalysts That Show Enhanced Activity for Carbon Dioxide Electrolysis. J. Phys. Chem. C.

[cit16] Rafiee M., Abrams D. J., Cardinale L., Goss Z., Romero-Arenas A., Stahl S. S. (2024). Cyclic voltammetry and chronoamperometry: mechanistic tools for organic electrosynthesis. Chem. Soc. Rev..

[cit17] Lehane R. A., Gamero-Quijano A., Manzanares J. A., Scanlon M. D. (2024). Mechanistic Insights into the Potentiodynamic Electrosynthesis of PEDOT Thin Films at a Polarizable Liquid|Liquid Interface. J. Am. Chem. Soc..

[cit18] Du X., Du A., Wang D., Mao Y., Zhang Z., Xie W. (2025). Surface-Enhanced Raman Spectroscopic Study of Key Intermediates in Electrochemical Ammonia Decomposition. J. Am. Chem. Soc..

[cit19] Zong C., Chen C.-J., Zhang M., Wu D.-Y., Ren B. (2015). Transient Electrochemical Surface-Enhanced Raman Spectroscopy: A Millisecond Time-Resolved Study of an Electrochemical Redox Process. J. Am. Chem. Soc..

[cit20] Singha Roy S., Nagappan S., Satheesan A. K., Karmakar A., Kundu S. (2024). Surface-Enhanced Raman Scattering Coupled with In Situ Raman Spectroscopy for the Detection of the OER Mechanism: A Mini-Review. J. Phys. Chem. C.

[cit21] Shan W., Liu R., Zhao H., He Z., Lai Y., Li S., He G., Liu J. (2020). In Situ Surface-Enhanced Raman Spectroscopic Evidence on the Origin of Selectivity in CO_2_ Electrocatalytic Reduction. ACS Nano.

[cit22] Bao H., Motobayashi K., Zhang H., Cai W., Ikeda K. (2023). In-situ Surface-Enhanced Raman Spectroscopy Reveals a Mars–van Krevelen-Type Gas Sensing Mechanism in Au@SnO_2_ Nanoparticle-Based Chemiresistors. J. Phys. Chem. Lett..

[cit23] Brosseau C. L., Colina A., Perales-Rondon J. V., Wilson A. J., Joshi P. B., Ren B., Wang X. (2023). Electrochemical surface-enhanced Raman spectroscopy. Nat. Rev. Methods Primers.

[cit24] Brown T. A., Chen H., Zare R. N. (2015). Identification of Fleeting Electrochemical Reaction Intermediates Using Desorption Electrospray Ionization Mass Spectrometry. J. Am. Chem. Soc..

[cit25] Brown T. A., Hosseini-Nassab N., Chen H., Zare R. N. (2016). Observation of electrochemically generated nitrenium ions by desorption electrospray ionization mass spectrometry. Chem. Sci..

[cit26] Liu J., Yu K., Zhang H., He J., Jiang J., Luo H. (2021). Mass spectrometric detection of fleeting neutral intermediates generated in electrochemical reactions. Chem. Sci..

[cit27] Chen J., Wang X., Cui X., Li Y., Feng Y., Wei Z. (2023). In Situ Probing and Identification of Electrochemical Reaction Intermediates by Floating Electrolytic Electrospray Mass Spectrometry. Angew. Chem., Int. Ed..

[cit28] Herl T., Matysik F.-M. (2020). Recent Developments in Electrochemistry–Mass Spectrometry. ChemElectroChem.

[cit29] Chang C.-W., Wehner D., Prabhu G. R. D., Moon E., Safferthal M., Bechtella L., Österlund N., Vos G. M., Pagel K. (2025). Elucidating reactive sugar-intermediates by mass spectrometry. Commun. Chem..

[cit30] Zhang X., Zhan J., Yu Z., Deng J., Li M., Shao Y. (2023). Recent Advances in Real-Time Analysis of Electrochemical Reactions by Electrochemical Mass Spectrometry. Chin. J. Chem..

[cit31] Bertani R., Michelin R. A., Mozzon M., Traldi P., Seraglia R., Busetto L., Cassani M. C., Tagliatesta P., D'Arcangelo G. (1997). Mass Spectrometric Detection of Reactive Intermediates. Reaction Mechanism of Diazoalkanes with Platinum(0) and Gold(I) Complexes. Organometallics.

[cit32] Kulesa K. M., Hirtzel E. A., Nguyen V. T., Freitas D. P., Edwards M. E., Yan X., Baker L. A. (2024). Interfacing High-Throughput Electrosynthesis and Mass Spectrometric Analysis of Azines. Anal. Chem..

[cit33] Wang T., Cheng Q., Hu J., Chen H., Xu J. (2023). Capture and identification of the electrogenerated picosecond intermediates by mass spectrometry. Fundam. Res..

[cit34] Freitas D., Chen X., Cheng H., Davis A., Fallon B., Yan X. (2021). Recent Advances of In-Source Electrochemical Mass Spectrometry. ChemPlusChem.

[cit35] Liu P., Lu M., Zheng Q., Zhang Y., Dewald H. D., Chen H. (2013). Recent advances of electrochemical mass spectrometry. Analyst.

[cit36] Hawkins B. C., Chalker J. M., Coote M. L., Bissember A. C. (2024). Electrochemically Generated Carbocations in Organic Synthesis. Angew. Chem., Int. Ed..

[cit37] Kumar A., Mondal S., Banerjee S. (2021). Aqueous Microdroplets Capture Elusive Carbocations. J. Am. Chem. Soc..

[cit38] Kumar A., Mondal S., Mofidfar M., Zare R. N., Banerjee S. (2022). Capturing Reactive Carbanions by Microdroplets. J. Am. Chem. Soc..

[cit39] Kumar A., Mondal S., Sandeep S., Venugopalan P., Kumar A., Banerjee S. (2022). Destabilized Carbocations Caged in Water Microdroplets: Isolation and Real-Time Detection of α-Carbonyl Cation Intermediates. J. Am. Chem. Soc..

[cit40] Kumar A., Mondal S., Banerjee S. (2023). Efficient Desorption and Capture of Reactive Carbocations from Positively Charged Glass Surface Bombarded with High-Speed Water Microdroplets. J. Phys. Chem. C.

[cit41] Banerjee S. (2023). On the stability of carbocation in water microdroplets. Int. J. Mass Spectrom..

[cit42] Nandy A., Kumar A., Mondal S., Koner D., Banerjee S. (2023). Spontaneous Generation of Aryl Carbocations from Phenols in Aqueous Microdroplets: Aromatic S_N_1 Reactions at the Air–Water Interface. J. Am. Chem. Soc..

[cit43] Jin Y., Petrovic P. V., Huang S., Banerjee S., Nandy A., Anastas P. T., Lam J. C.-H. (2024). Carbocation Mechanism Revelation of Molecular Iodine-Mediated Dehydrogenative Aromatization of Substituted Cyclic Ketones to Phenols. J. Org. Chem..

[cit44] Hou Z.-W., Liu D.-J., Xiong P., Lai X.-L., Song J., Xu H.-C. (2021). Site-Selective Electrochemical Benzylic C–H Amination. Angew. Chem., Int. Ed..

[cit45] Meng L., Su J., Zha Z., Zhang L., Zhang Z., Wang Z. (2013). Direct Electrosynthesis of Ketones from Benzylic Methylenes by Electrooxidative C–H Activation. Chem.–Eur. J..

[cit46] Wang H., Liang K., Xiong W., Samanta S., Li W., Lei A. (2020). Electrochemical oxidation-induced etherification via C(sp^3^)–H/O–H cross-coupling. Sci. Adv..

[cit47] Zhang Y., Hou J., Yang H., Wang S., Yuan K. (2023). Electrochemically enhanced deoxygenative cross-coupling of aryl ketones with heteroarenes through in situ generated benzyl carbocations. Org. Biomol. Chem..

[cit48] Yang Y.-Z., Wu Y.-C., Song R.-J., Li J.-H. (2020). Electrochemical dehydrogenative cross-coupling of xanthenes with ketones. Chem. Commun..

[cit49] Shao X., Tian L., Wang Y. (2019). C–N Coupling of Azoles or Imides with Carbocations Generated by Electrochemical Oxidation. Eur. J. Org. Chem..

[cit50] Hong J. E., Yoon J., Baek W., Kim K., Kwak J.-H., Park Y. (2023). Electrochemical C(sp^3^)–H Lactonization of 2-Alkylbenzoic Acids toward Phthalides. Org. Lett..

[cit51] Yan X. (2021). Emerging microdroplet chemistry for synthesis
and analysis. Int. J. Mass Spectrom..

[cit52] Banerjee S., Gnanamani E., Yan X., Zare R. N. (2017). Can all bulk-phase reactions be accelerated in microdroplets?. Analyst.

[cit53] Banerjee S., Prakash H., Mazumdar S. (2011). Evidence of Molecular Fragmentation inside the Charged Droplets Produced by Electrospray Process. J. Am. Soc. Mass Spectrom..

[cit54] Lee J. K., Banerjee S., Nam H. G., Zare R. N. (2015). Acceleration of reaction in charged microdroplets. Q. Rev. Biophys..

[cit55] Girod M., Moyano E., Campbell D. I., Cooks R. G. (2011). Accelerated bimolecular reactions in microdroplets studied by desorption electrospray ionization mass spectrometry. Chem. Sci..

[cit56] Wang T., Li Z., Gao H., Hu J., Chen H.-Y., Xu J.-J. (2023). Ultrafast C–C and C–N bond formation reactions in water microdroplets facilitated by the spontaneous generation of carbocations. Chem. Sci..

[cit57] MacDonald T. S. C., Price W. S., Beves J. E. (2019). Time-Resolved Diffusion NMR Measurements for Transient Processes. ChemPhysChem.

