# Supplementary material for: Seeing the unseen: spatio-temporal visualization of reactive carbocation intermediates in electrolytic cells
Source: Chem Sci. 2025 Oct 6;16(44):21020–7. doi: 10.1039/d5sc06447c (PMC12516860; doi:10.1039/d5sc06447c)
Supplement: SC-016-D5SC06447C-s001 [file SC-016-D5SC06447C-s001.pdf]

Supplementary Information (SI) for Chemical Science.
This journal is © The Royal Society of Chemistry 2025

Supplementary Information

Seeing the Unseen: Spatio-temporal Visualization of Reactive Carbocation Intermediates in Electrolytic Cells

Abhijit Nandy,[§] Barsha Pathak,[§] Bikash Ranjan Isaac, Vijayamohanan Pillai,[*] and Shibdas Banerjee[*]

Department of Chemistry, Indian Institute of Science Education and Research Tirupati, Tirupati 517507, India

*Corresponding authors:

Vijayamohanan Pillai, E-mail address: vijay@iisertirupati.ac.in

Shibdas Banerjee, E-mail address: shibdas@iisertirupati.ac.in

[§]Contributed equally

Sr. No.	***Contents***	***Page No.***
1	Materials and Methods	S2-S5
2	Supplementary Note 1	S6
3	Table S1	S7-S8
4	Fig. S1	S9
5	Fig. S2	S10
6	Fig. S3	S11
7	Fig. S4	S12
8	Fig. S5	S13
9	Fig. S6	S14
10	Fig. S7	S15
11	Fig. S8	S16
12	Fig. S9	S17
13	Fig. S10	S18
14	Fig. S11	S19
15	Fig. S12	S20
16	References	S21

Materials and Methods

All required chemicals were acquired from Tokyo Chemical Industry (Japan) and Sigma-Aldrich (St. Louis, MO). UHPLC-MS grade solvents, including deionized water (resistivity ≥18.2 MΩ cm at 298 K), were purchased from Fischer Chemicals (Waltham, Massachusetts, USA). Microscope glass slides and other necessary glassware were obtained from Borosil Glass Works Ltd (Ahmedabad, India). Stainless steel tubing, fused silica tubing, capillary tubing sleeves, union bodies, connectors, and Hamilton syringes were sourced from IDEX Corporation (Illinois, USA).

Desorption electrospray ionization mass spectrometry (DESI-MS)

A custom-built DESI source connected to a high-resolution mass spectrometer (Orbitrap Exploris 120, ThermoFisher Scientific, NH, USA) was used for the DESI-MS investigations. The design of the source was similar to that used in our previous work.[1-4] The source was designed with an outside (coaxial) stainless steel capillary (0.5 mm i.d. and 1.6 mm o.d.) for nebulizing gas (nitrogen) supply and an inner fused silica capillary (100 µm i.d. and 360 µm o.d.) for solvent delivery. Charged microdroplets were generated in positive mode by applying a +5 kV potential to the stainless-steel needle of the solvent syringe. The LC-MS grade water was pumped through the inner silica tubing at a rate of 10 µL/min, with a coaxial sheath gas of nitrogen supplied at a back pressure of 110 psi. A stream of charged microdroplets was produced using the DESI source at ambient temperature and atmospheric pressure. This stream was directed to impact a standard microscope glass slide at an incident angle of 55°, with a spray tip-to-surface distance of 5 mm, a spray tip-to-mass spectrometer inlet distance of approximately 10 mm, and a collection angle of about 5°. Using these geometrical parameters, a DESI spray spot with a diameter of about 1 mm was formed on the glass slide using water. While the MS was in use for data collection, a Hamilton syringe was used to rapidly dispense a reaction aliquot of 10 µL onto the microscope glass slide in the impinging spray of charged microdroplets. These charged microdroplets splashed, creating secondary microdroplets that extracted the analyte molecules (ions). The analyte ions were then completely desolvated and transported to the mass spectrometer via a heated capillary inlet (stainless steel, length 14.2 cm, i.d. 550 µm). The S-lens RF level was set to 70%, and the heated capillary (MS inlet) temperature was kept at 300 °C. Unless specified otherwise, all experiments were performed using the same setup to identify species within the *m/z* range of 40–600 at a mass resolution of 120,000. To maximize ion count, other ion optics parameters were optimized. Data acquisition was carried out using XCalibur software (Thermo Fisher Scientific). Species identification was based on their isotope distribution patterns and high mass accuracy (error <5 ppm).

Electrochemical reaction sampling

The electrochemical reaction chamber consisted of a 50 mL glass beaker (inner diameter: 45 mm) sealed with a plastic lid, through which the cathode and anode were inserted by puncturing the lid (Fig. S1).

The electrodes were positioned in the center of the chamber, separated by a distance of 18 mm. A total of 21 predefined sampling ports (small holes) were made on the lid to collect liquid samples (reaction aliquots) from various positions along the chamber, relative to the anode. The reaction volume was maintained at 12 mL to ensure that the electrodes remained submerged by approximately 0.5 cm, enabling efficient electrochemical reaction. The electroorganic synthesis was carried out in the chamber using a Keithley 2200 DC power supply, operated at the desired current setting.

For temporal analysis of the electrochemical reaction, 10 µL aliquots were periodically withdrawn using a Hamilton syringe needle, which was vertically inserted through the sampling port located 3 mm from the anode (toward the cathode side) at a depth aligned with the center of the anode, and immediately analyzed by DESI-MS.

Analogously, for spatial analysis of the electrochemical reaction, the Hamilton syringe needle was vertically inserted through each sampling port to a typical depth aligned with the center of the anode to withdraw 10 µL aliquots for DESI-MS analysis. In total, 21 such aliquots were collected and analyzed from different locations across the chamber. All sampling and analysis were completed within approximately 3.5 minutes (10 seconds per sample as optimized in our earlier studies) during the reaction period when carbocation abundance was known to be sufficiently high, based on prior temporal profiling. We also confirmed that the variation in carbocation abundance remained minimal within this time frame, as demonstrated by temporal profiling (e.g., the time interval highlighted in yellow in Fig. 3c). This ensured that any spatio-temporal fluctuation in analyte abundance during sampling remained within experimental error range and did not impact the overall conclusions of the study. The absolute abundance of the carbocation intermediate observed in the mass spectra was mapped to the corresponding spatial coordinates of the sampling ports, enabling the construction of an interpolated contour plot representing the carbocation intensity distribution. All results were validated by altering the sequence of spatial sampling across at least three independent experiments. Among these, only one representative interpolated contour plot (*e.g.,* Fig. 3d) illustrating the spatial distribution of carbocation abundance is presented in this study, as all three sets yielded overall reproducible and consistent results.

Electrochemical syntheses involving carbocation intermediates

Various types of carbocation intermediates were produced via model electrochemical reactions (see below) and subsequently intercepted and identified using DESI-MS.

Site-selective electrochemical benzylic C-H amination (Fig. 2a-c)

We followed the report of Hou et al.[5] (Fig. 2a-c) to perform this reaction. Arenes (0.3 mmol), para-toluenesulphonamide (0.6 mmol), and nBu_4NBF_4 (0.36 mmol) were introduced into an electrochemical reaction chamber fitted with a platinum plate cathode and a reticulated vitreous carbon (RVC) anode. The chamber was flushed with argon, followed by sequential addition of dichloroethane (DCE, 8 mL)

and hexafluoroisopropanol (HFIP, 4 mL) as solvents. A 7.5 mA constant current was maintained throughout the reaction. During the course of the reaction, 10 µL aliquots were periodically sampled for DESI-MS study.

Direct electrosynthesis of ketones from benzylic methylenes by electrooxidative C-H activation (Fig. 2b)

We followed the report of Meng et al.[6] (Fig. 2b) to perform this reaction. An undivided electrochemical cell was charged with diphenylmethane (0.5 mmol), $LiClO_4$ (1 mmol), and a solvent mixture of acetonitrile (ACN, 9 mL) and water (0.5 mL). Two platinum plates served as the cathode and anode, respectively. Electrolysis was carried out at room temperature with a steady current of 20 mA. Throughout the reaction, 10 µL aliquots were periodically withdrawn for analysis by DESI-MS.

Electrochemical oxidation-induced etherification via C(sp3)-H/O-H cross-coupling (Fig. 2c)

This reaction was carried out following the procedure reported by Wang et al.[7] (Fig. 2c). Cs_2CO_3 (1 mmol) and $^nBu_4NClO_4$ (0.25 mmol) were added to an electrochemical reaction chamber. A carbon rod was used as the anode, while a nickel plate served as the cathode. Sequentially DCE (4 ml), indan (0.5 mmol), *t*-butyl alcohol (1.5 mmol), and Et_2O (0.5 ml) were added. For 3.5 hours, the electrolysis was conducted at a continuous current of 10 mA at a room temperature of 20°C. During the course of the reaction, 10 µL aliquots were periodically sampled for DESI-MS study.

Deoxygenative cross-coupling (Fig. 2d)

This reaction was carried out following the procedure reported by Zhang et al.[8] (Fig. 2d). The experiment was conducted in an electrochemical reaction chamber equipped with a graphite anode and a copper cathode. Benzophenone (0.5 mmol), triethylsilane (0.25%), anisole (1 mmol), acetonitrile (10 mL), and trifluoromethanesulfonic acid (0.9 mmol) were sequentially introduced into the chamber. Electrolysis was performed at room temperature under a constant current of 10 mA. During the course of the reaction, 10 µL aliquots were periodically sampled for DESI-MS study.

C-N Coupling of Azoles (Fig. 2e)

The reaction was performed according to the protocol described by Shao et al.[9] (Fig. 2e). A carbon electrode served as the anodic electrode and a nickel plate served as the cathodic electrode in an electrochemical reaction chamber. It was then filled with ACN (10 ml), $^nBu_4NClO_4$ (0.2 mmol), xanthene (0.9 mmol), and imidazole (0.3 mmol). For 3.4 hours, the reaction mixture was electrolyzed at room temperature at a constant current of 7 mA. Throughout the reaction, 10 µL aliquots were periodically withdrawn for analysis by DESI-MS.

Dehydrogenative cross-coupling of xanthenes with ketones (Fig. 2f)

We followed the report of Yang et al.[10] (Fig. 2f) to perform this reaction. Xanthene (0.25 mmol), ketones (2 equiv.), nBu_4NBF_4 (0.4 mmol), and ACN (10 mL) were combined and introduced into an oven-dried electrochemical reaction chamber. Methanesulfonic acid (MsOH, 2 equiv.) was then added to the mixture. A platinum plate was employed as the cathode and a graphite electrode as the anode. The reaction was carried out at a constant current of 5 mA for 2 hours. During the course of the reaction, 10 μL aliquots were periodically sampled for DESI-MS study.

C(sp^3)–H Lactonization (Fig. 2g):

We followed the report of Hong et al.[11] (Fig. 2g) to perform this reaction. A nickel plate served as the cathode and a graphite electrode served as the anode in an electrochemical reaction chamber. 2,4-dimethylbenzoic acid (0.5 mmol), $^{n}Bu_4NClO_4$, dichloromethane (DCM, 8 ml), and HFIP (2 ml) were then added to it. The reaction mixture was electrolyzed for three hours at room temperature at a current of 15 mA. Throughout the reaction, 10 μL aliquots were periodically withdrawn for analysis by DESI-MS.

Supplementary Note 1

Unlike conventional chemical methods that rely on stoichiometric oxidants or reductants, electroorganic synthesis harnesses electron transfer to initiate and control reactions, making it an inherently green and atom-economical strategy. Electroorganic reactions are fundamentally driven by electrochemical activation, wherein reactants experience direct electron transfer at the electrode interface. This process often generates key reactive species such as radicals, radical cations, carbocations, or carbanions, which subsequently participate in bond-forming reactions. In situ identification of such reactive species is crucial for deducing the mechanistic details of an electrochemical transformation. Traditionally, cyclic voltammetry (CV) has been extensively employed as an electroanalytical technique to study redox potentials and kinetics.[12-14] However, it solely measures current responses without providing molecular-level identification of intermediates, thereby limiting its ability to distinguish between multiple reactive species present in the reaction medium. Furthermore, the challenges posed by overlapping redox peaks, signal averaging, interference from side reactions, and the limited sensitivity of cyclic voltammetry to transient or non-electroactive intermediates underscore the necessity for complementary techniques that enable real-time monitoring of reactive intermediates in electrochemical processes. Fourier-transform infrared (FTIR) spectroscopy and surface-enhanced Raman spectroscopy (SERS) have also been employed to study electrochemical intermediates. However, FTIR has low sensitivity to short-lived species and is prone to solvent interference, while SERS is limited to interfacial species, failing to capture bulk-phase intermediates, restricting comprehensive mechanistic insights.[15-16] As a result, mechanistic insights in electroorganic synthesis often remain speculative, creating a significant disconnect between proposed pathways and experimental confirmation. Direct detection and characterization of short-lived electrochemical intermediates are essential to close this gap.

Table S1. A list of precursor compounds along with the corresponding radical cations and carbocations generated through electrochemical reactions. The *m/z* values and the mass accuracies of these charged species are also provided in the table.*

Precursor Substrate	Intermediates	Theoretical *m/z*	Observed *m/z*	Error (ppm)
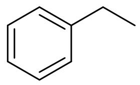	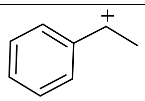	105.0699	105.0700	0.95
	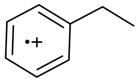	106.0778	106.0779	0.94
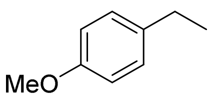	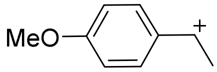	135.0804	135.0804	0
	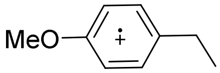	136.0883	136.0883	0
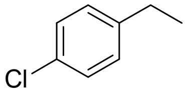	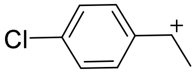	139.0309	139.0306	-2.15
	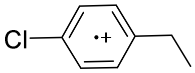	140.0388	140.0385	-2.14
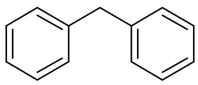	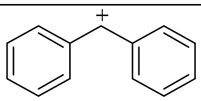	167.0855	167.0853	-1.19
	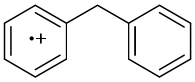	168.0934	168.0932	-1.18
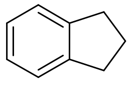	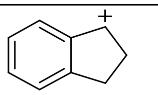	117.0699	117.0697	-1.70
	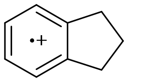	118.0778	118.0776	-1.69
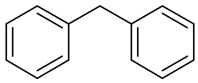	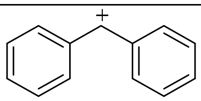	167.0855	167.0854	-0.59
	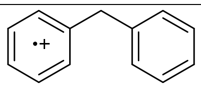	168.0934	168.0933	-0.59
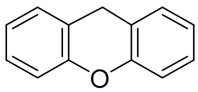	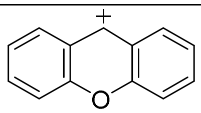	181.0648	181.0647	-0.55
	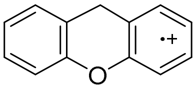	182.0727	182.0725	-1.10

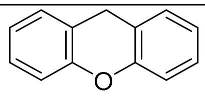	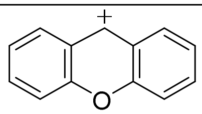	181.0648	181.0646	-1.10
	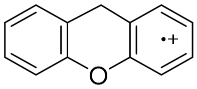	182.0727	182.0724	-1.64
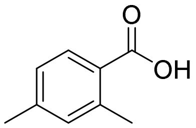	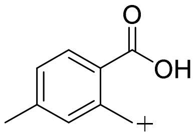	145.0597	149.0596	-0.69

*Variations in mass (*m/z*) accuracy are attributed to changes in the calibration of the orbitrap analyzer on different experimentation days. Nonetheless, an overall *m/z* accuracy of <5 ppm was maintained throughout the experiments. It is worth noting that slight differences in the observed *m/z* values at the fourth decimal place may appear in other parts of this study; however, these variations remain within the specified accuracy range (<5 ppm).

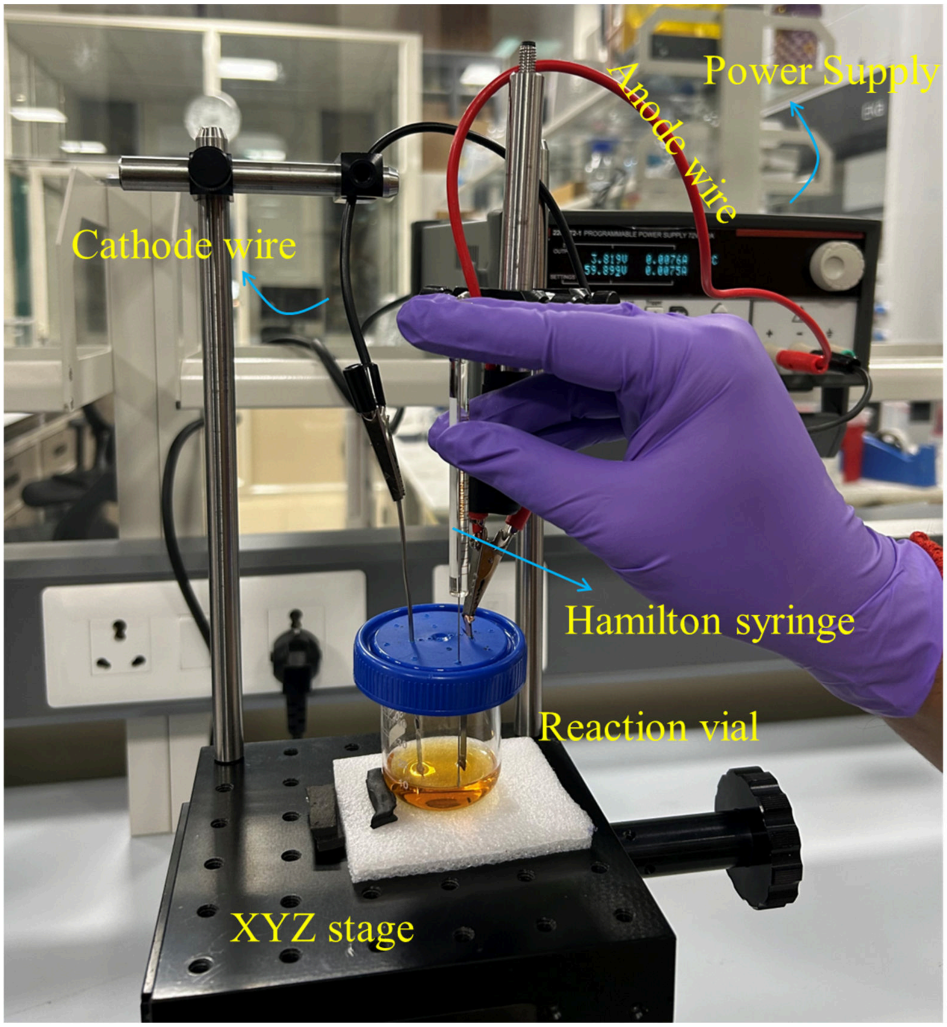

Fig. S1. A representative photograph of the experimental setup showing the collection of an electrochemical reaction aliquot through a typical sampling port (hole) using a Hamilton syringe for subsequent DESI-MS analysis. Several predefined sampling ports (holes) were created into the lid at varying distances from the anode to enable spatially resolved sampling of the reaction. See more details in the Materials and Methods.

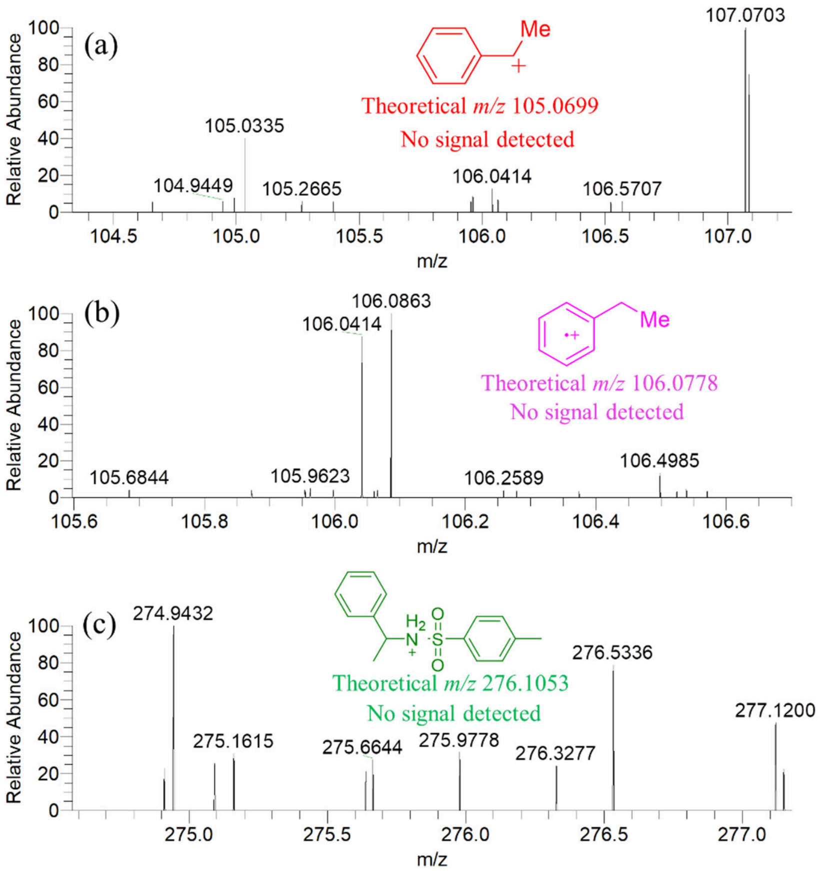

Fig. S2. Positive ion mode DESI-MS was recorded from the control experiment with the precursor substrate solution (**1a** in Fig. 2a) without starting the electrochemical reaction (no current applied). The precursor solution, maintained at a similar concentration in the corresponding solvent required for the reaction, was delivered to the DESI source under the impinging spray of charged aqueous microdroplets, followed by mass spectral analysis. No ion signals were observed for the carbocation (*m/z* 105.0699) (a), radical cation (*m/z* 106.0778) (b), or product (*m/z* 276.1053) (c). The detected signals (unassigned) are attributed to either blank background noise and/or artifacts/impurities present in the commercially available substrate.

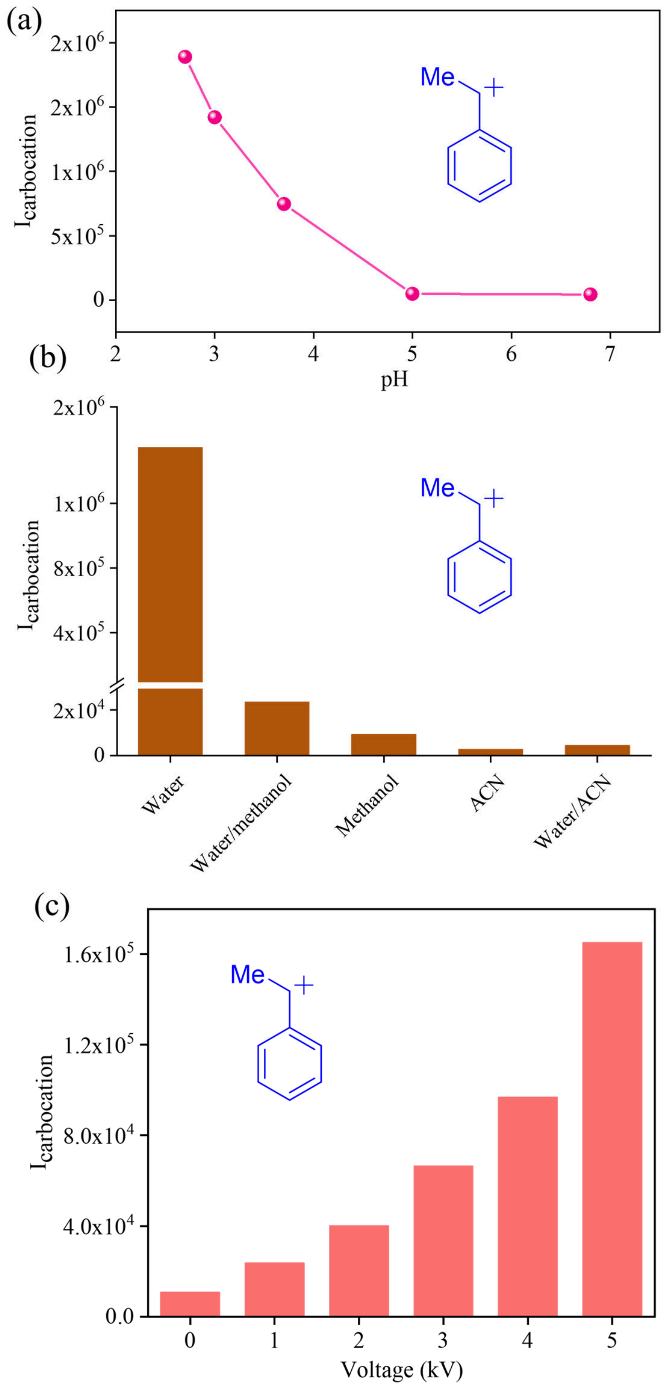

Fig. S3. Plots showing the intensity of the benzylic carbocation (inset) signal as a function of (a) pH of water used in the DESI spray, (b) different sprayed solvents, and (c) varying spray potential in the DESI source for intercepting the carbocation from the electrochemical reaction aliquot (Fig. 2a). pH and solvent dependent study were examined at a spray potential of +5 kV. The reaction mixture was aliquoted at 20 min of the reaction age for pH, solvent, and voltage dependence studies. The pH was adjusted from 6.8 to 2.15 by adding formic acid to the water. A total of eleven electrochemical reactions were conducted—five for the pH study, five for the solvent-dependent study, and one for the voltage-dependent study, with LC-MS grade water (pH 6.8) serving as the common condition for the solvent- and voltage-dependent experiments.

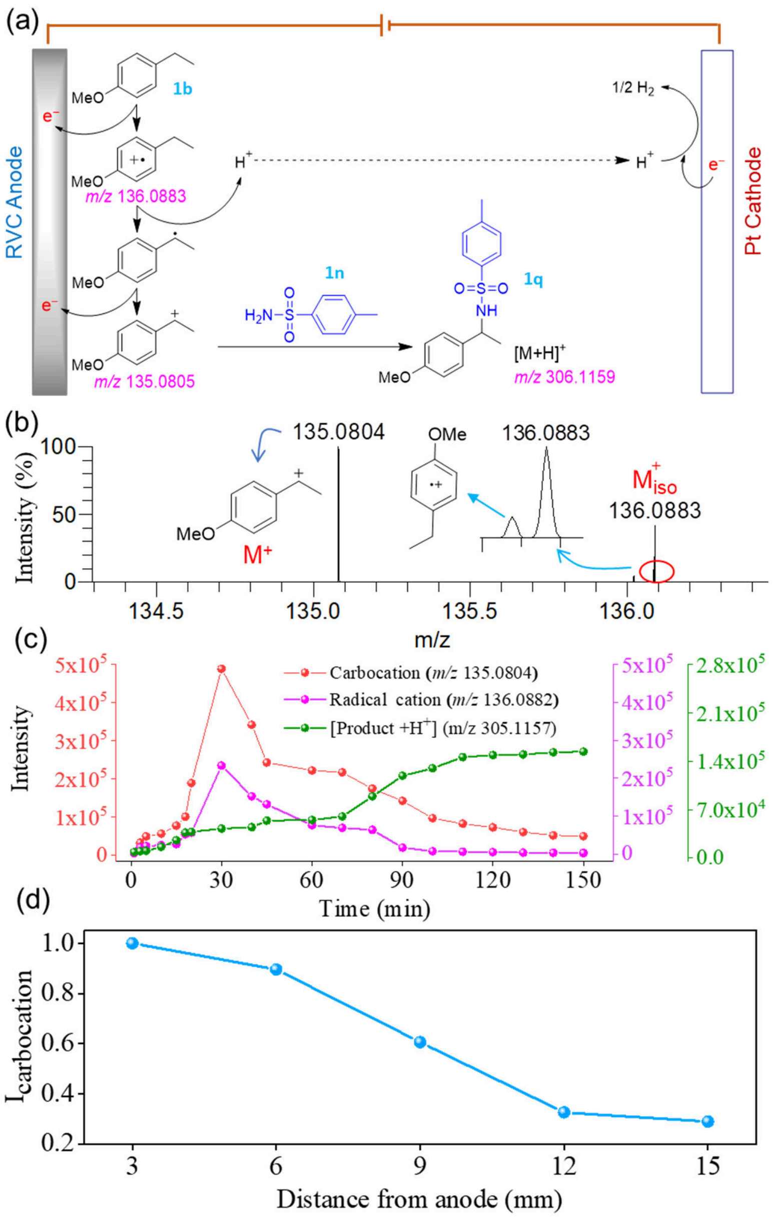

Fig. S4. (a). Schematic presentation of carbocation intermediacy in the site-selective electrochemical benzylic C-H activation of 1-ethyl-4-methoxybenzene (**1b** in Fig. 2a) with a plausible mechanism.[5] (b) DESI-MS detection of the 1-(4-methoxyphenyl)ethan-1-ylium carbocation intermediate involved in the reaction. (c) Temporal evolution of the sequential formation of the intermediate radical cation, carbocation, and the product in the electrochemical reaction, enabling their real-time monitoring. (d) Normalized plot showing the decrease in carbocation abundance (measured at the depth aligned with the center of the anode) in the electrolytic cell as the sampling location (for DESI-MS) moves from the anode to the cathode. For this spatial profiling of carbocation, the reaction mixture was sequentially aliquoted for DESI-MS in the direction from the cathode to the anode in between 30 to 31 min of the reaction age (see panel c). The mass accuracies of the detected intermediate species are listed in Table S1.

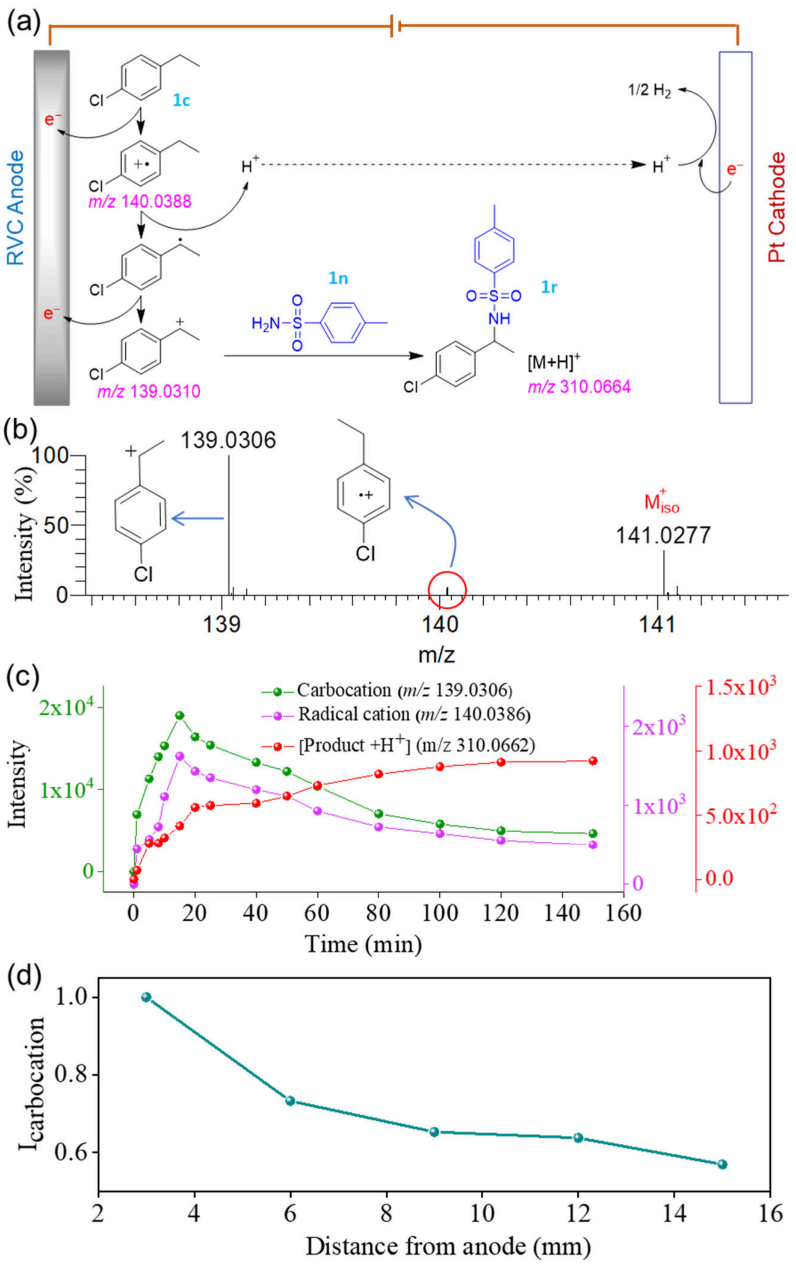

Fig. S5. a) Schematic presentation of carbocation intermediacy in the site-selective electrochemical benzylic C-H activation of 1-ethyl-4-chlorobenzene (**1c** in Fig. 2a) with a plausible mechanism.[5] (b) DESI-MS detection of the 1-(4-chlorophenyl)ethan-1-ylium carbocation intermediate involved in the reaction. (c) Temporal evolution of the sequential formation of the intermediate radical cation, carbocation, and the product in the electrochemical reaction, enabling their real-time monitoring. (d) Normalized plot showing the decrease in carbocation abundance (measured at the depth aligned with the center of the anode) in the electrolytic cell as the sampling location (for DESI-MS) moves from the anode to the cathode. For this spatial profiling of carbocation, the reaction mixture was sequentially aliquoted for DESI-MS in the direction from the cathode to the anode in between 15 to 16 min of the reaction age (see panel c). The mass accuracies of the detected intermediate species are listed in Table S1.

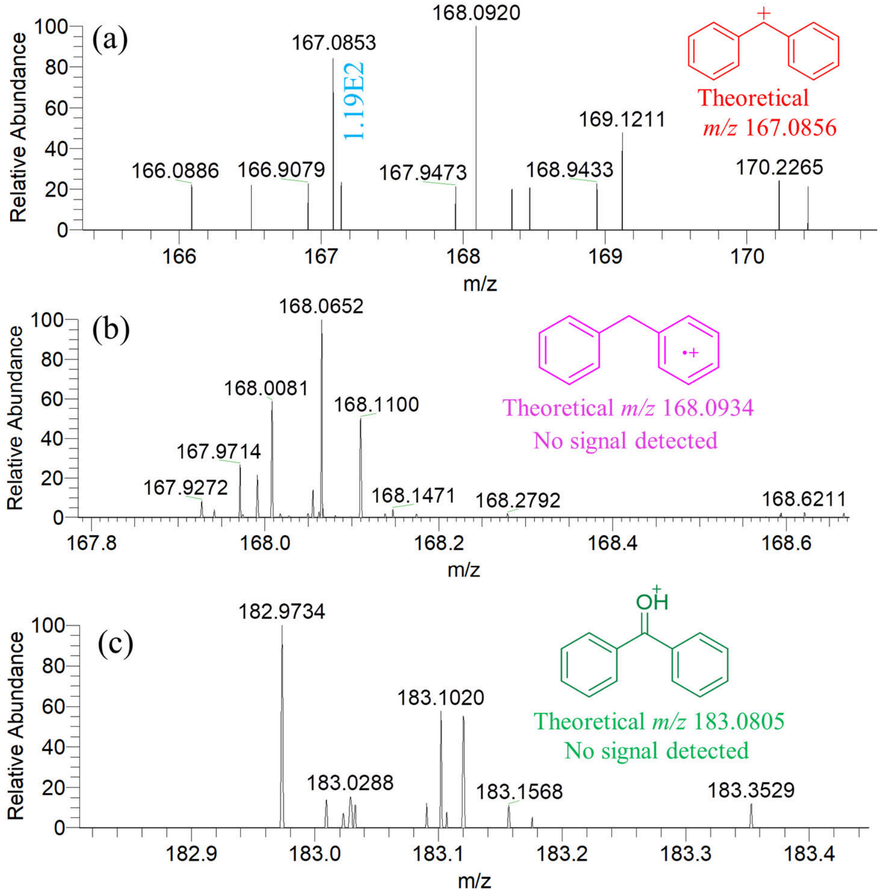

Fig. S6. Positive ion mode DESI-MS was recorded from the control experiment with the precursor substrate solution (**2a** in Fig. 2b) without starting the electrochemical reaction (no current applied). The precursor solution, maintained at a similar concentration in the corresponding solvent required for the reaction, was delivered to the DESI source under the impinging spray of charged aqueous microdroplets, followed by mass spectral analysis. Very trace ion signal was observed for the carbocation (*m/z* 167.0856, possibly due to microdroplet reaction) (a), whereas no detectable ion signals were observed for the radical cation (*m/z* 168.0934) (b) or the product (*m/z* 183.0805) (c). The unassigned signals are attributed to either blank background noise and/or artifacts/impurities present in the commercially available substrate. It should be noted that although a trace-level generation of carbocations in microdroplets is evident here (a), the pronounced temporal variation of this intermediate at appreciable intensities during the course of the reaction clearly establishes its role as a true electrochemical intermediate (Fig. 4c), with negligible or no contribution from microdroplet effects.

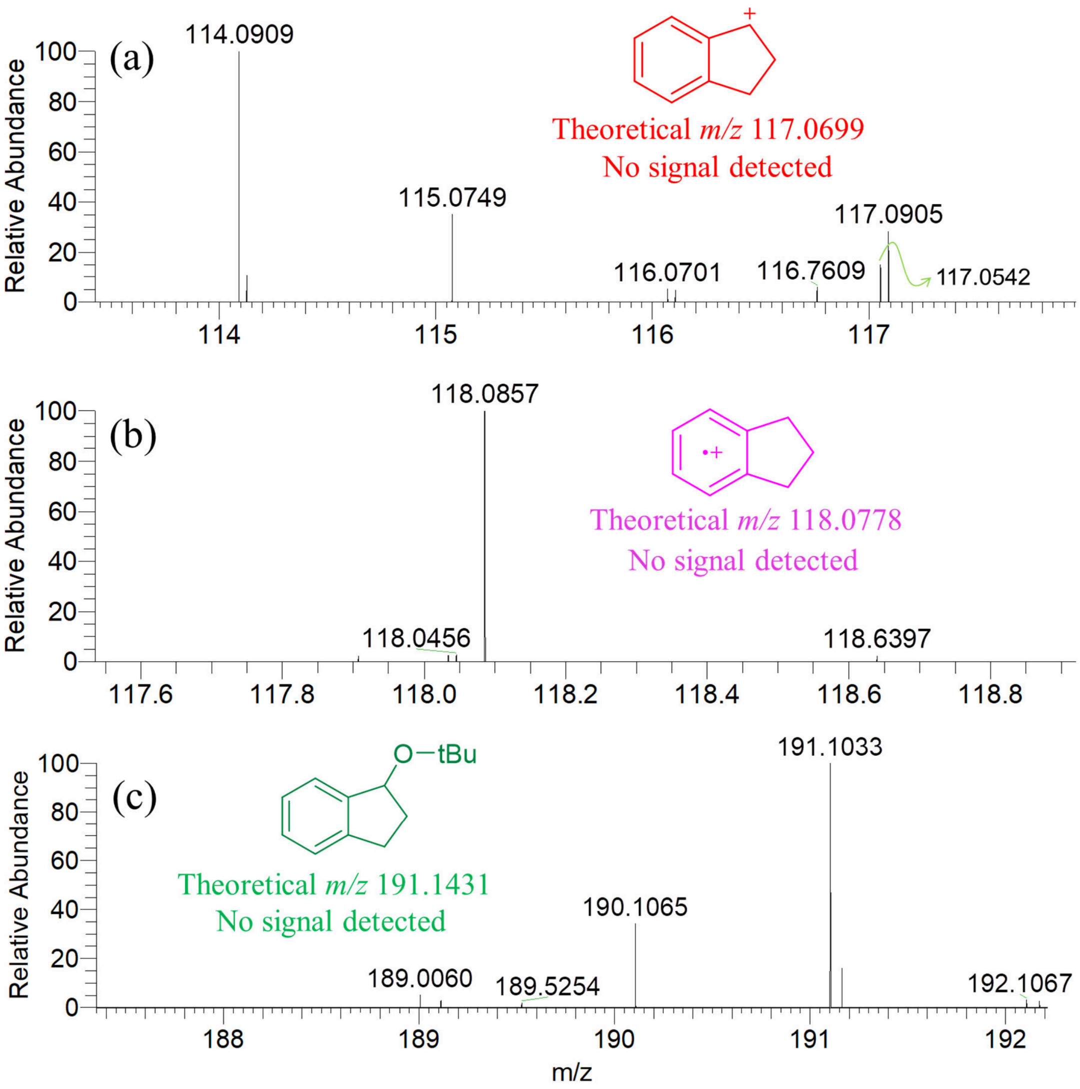

Fig. S7. Positive ion mode DESI-MS was recorded from the control experiment with the precursor substrate solution (**3a** in Fig. 2c) without starting the electrochemical reaction (no current applied). The precursor solution, maintained at a similar concentration in the corresponding solvent required for the reaction, was delivered to the DESI source under the impinging spray of charged aqueous microdroplets, followed by mass spectral analysis. No detectable ion signals were observed for the carbocation (*m/z* 117.0699) (a), radical cation (*m/z* 118.0778) (b), or product (*m/z* 191.1431) (c). The detected signals (unassigned) were attributed to either blank background noise and/or artifacts/impurities present in the commercially available substrate.

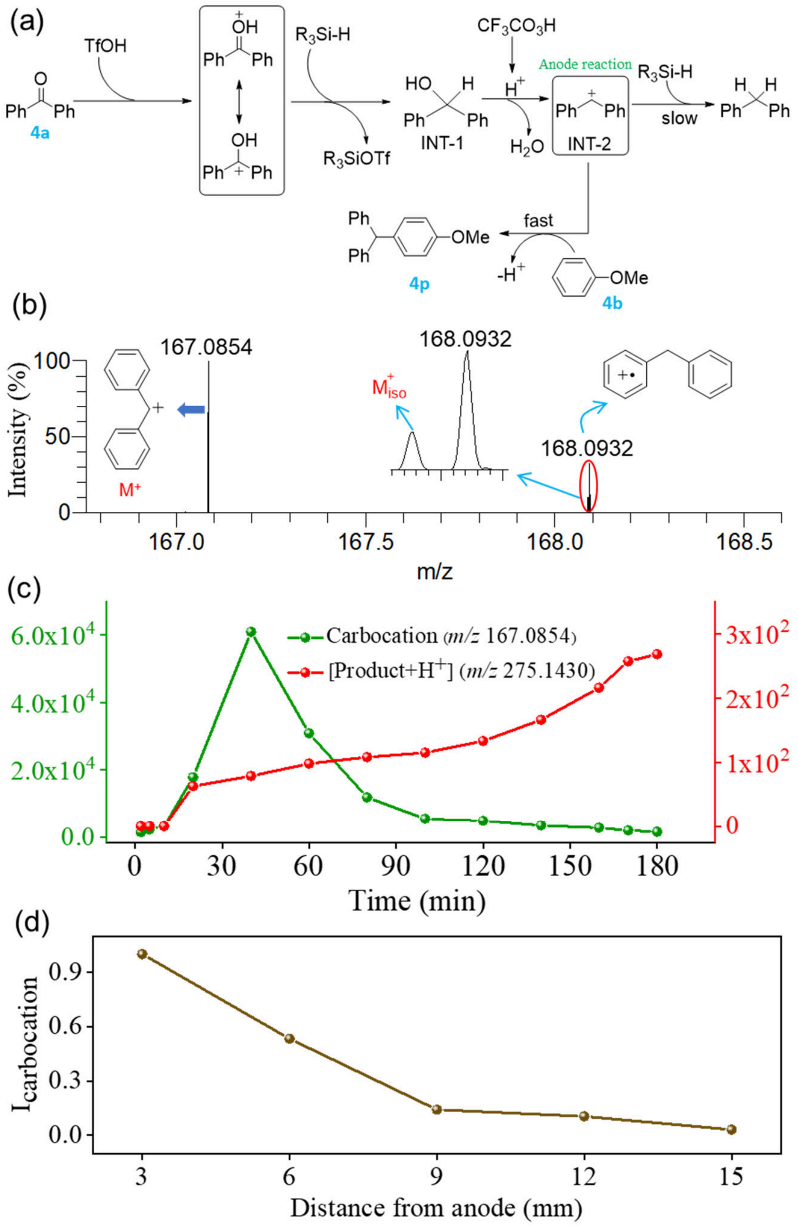

Fig. S8. a) Schematic presentation of carbocation intermediacy in the site-selective electrochemical deoxygenative cross coupling reaction of benzophenone (**4a** in Fig. 2d) with a plausible mechanism.[8] (b) DESI-MS detection of the diphenylmethylium carbocation intermediate involved in the reaction. (c) Temporal evolution of the sequential formation of the intermediate carbocation, and the product in the electrochemical reaction, enabling their real-time monitoring. (d) Normalized plot showing the decrease in carbocation abundance (measured at the depth aligned with the center of the anode) in the electrolytic cell as the sampling location (for DESI-MS) moves from the anode to the cathode. For this spatial profiling of carbocation, the reaction mixture was sequentially aliquoted for DESI-MS in the direction from the cathode to the anode in between 40 to 41 min of the reaction age (see panel c). The mass accuracies of the detected intermediate species are listed in Table S1.

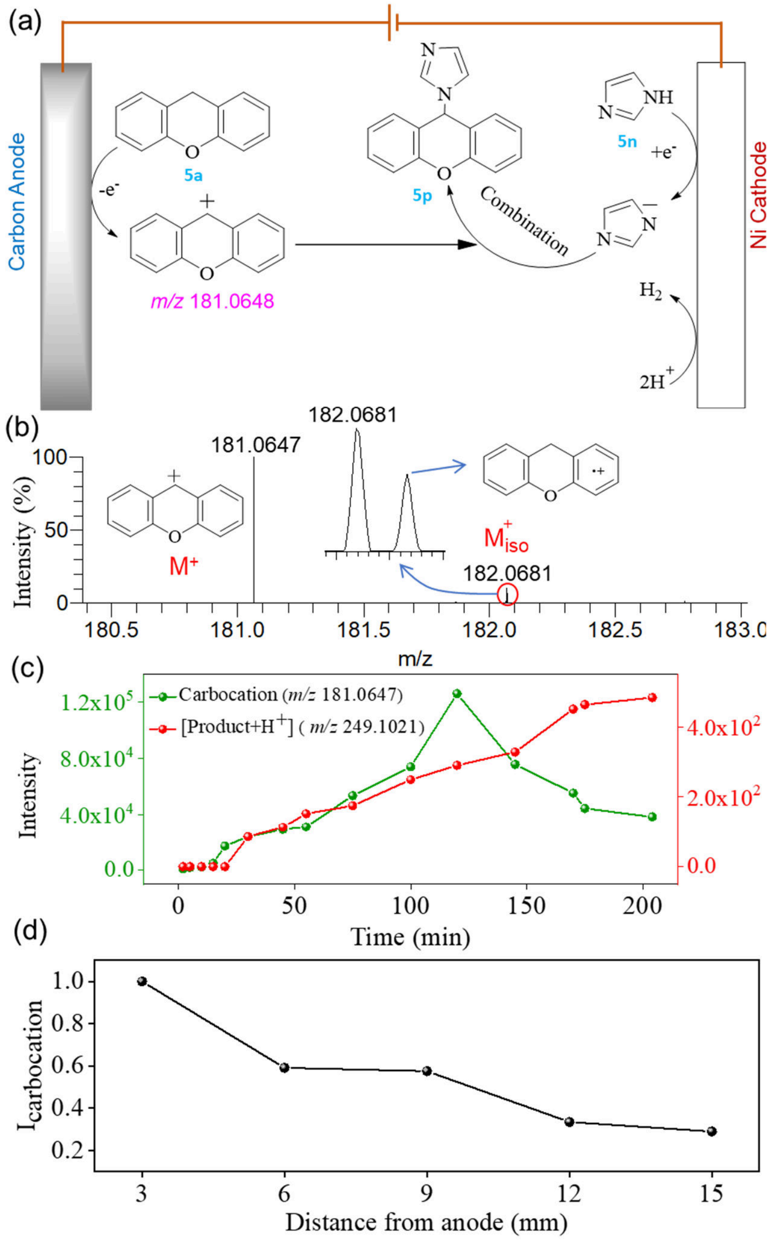

Fig. S9. a) Schematic presentation of carbocation intermediacy in the site-selective electrochemical C-N coupling reaction of xanthene and imidazole (**5a** in Fig. 2e) with a plausible mechanism.[9] (b) DESI-MS detection of the 9H-xanthen-9-ylium carbocation intermediate involved in the reaction. (c) Temporal evolution of the sequential formation of the intermediate carbocation, and the product in the electrochemical reaction, enabling their real-time monitoring. (d) Normalized plot showing the decrease in carbocation abundance (measured at the depth aligned with the center of the anode) in the electrolytic cell as the sampling location (for DESI-MS) moves from the anode to the cathode. For this spatial profiling of carbocation, the reaction mixture was sequentially aliquoted for DESI-MS in the direction from the cathode to the anode in between 120 to 121 min of the reaction age (see panel c). The mass accuracies of the detected intermediate species are listed in Table S1.

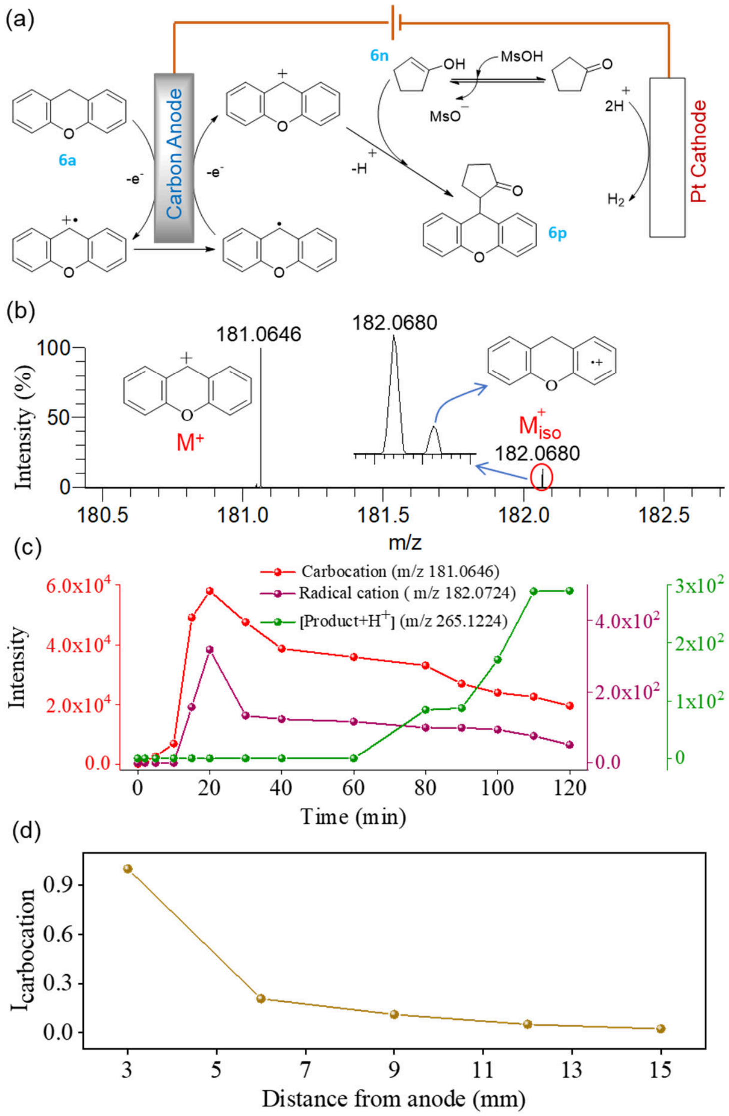

Fig. S10. a) Schematic presentation of carbocation intermediacy in the site-selective electrochemical dehydrogenative cross coupling reaction of xanthene and cyclopentanone (Fig. 2f) with a plausible mechanism.[10] (b) DESI-MS detection of the 9H-xanthen-9-ylium carbocation intermediate involved in the reaction. (c) Temporal evolution of the sequential formation of the intermediate radical cation, carbocation, and the product in the electrochemical reaction, enabling their real-time monitoring. (d) Normalized plot showing the decrease in carbocation abundance (measured at the depth aligned with the center of the anode) in the electrolytic cell as the sampling location (for DESI-MS) moves from the anode to the cathode. For this spatial profiling of carbocation, the reaction mixture was sequentially aliquoted for DESI-MS in the direction from the cathode to the anode in between 20 to 21 min of the reaction age (see panel c). The mass accuracies of the detected intermediate species are listed in Table S1.

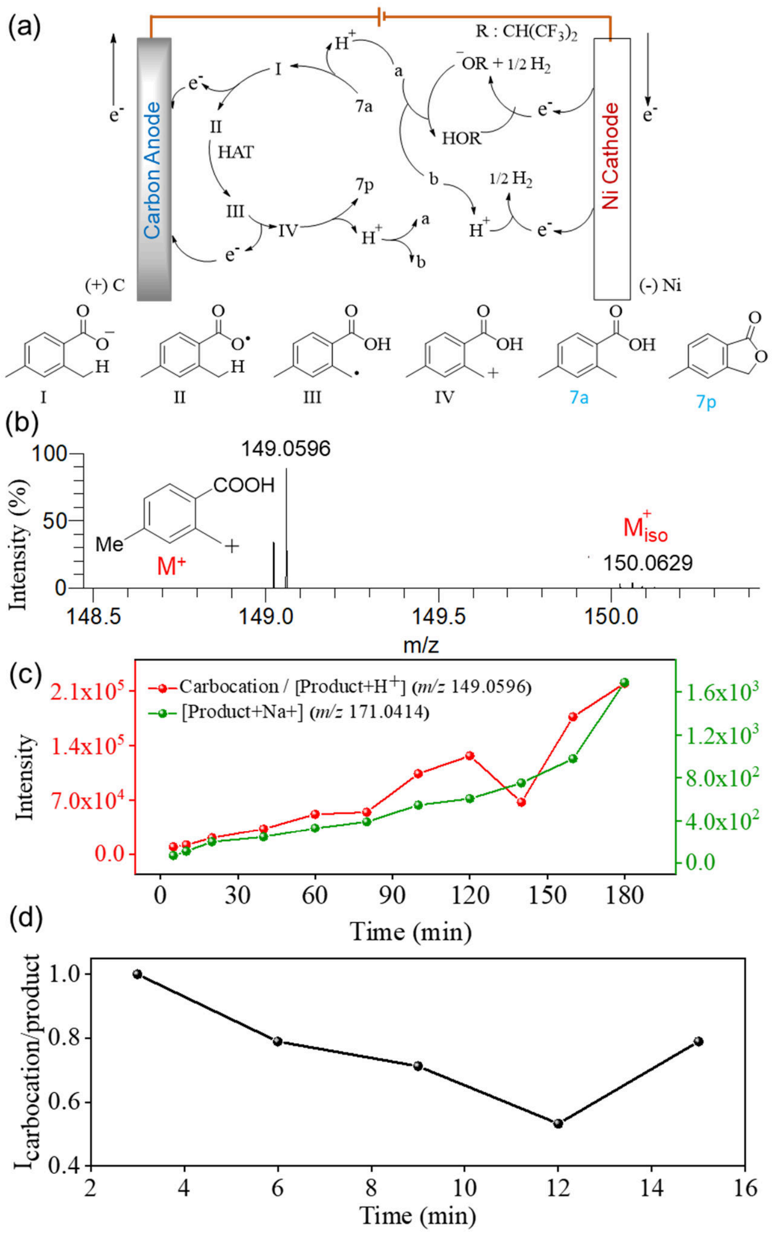

Fig. S11. a) Schematic presentation of carbocation intermediacy in the electrochemical C(sp3)–H lactonization of 2,4-dimethylbenzoic acid (**7a** in Fig. 2g) with a plausible mechanism.[11] (b) DESI-MS detection of the (2-carboxy-5-methylphenyl)methylium carbocation carbocation intermediate involved in the reaction. (c) Temporal evolution of the formation of the intermediate carbocation, and the product (isomeric with the carbocation) in the electrochemical reaction, enabling their real-time monitoring. (d) Normalized plot showing the decrease in carbocation and the isomeric product abundance (measured at the depth aligned with the center of the anode) in the electrolytic cell as the sampling location (for DESI-MS) moves from the anode to the cathode. For this spatial profiling, the reaction mixture was sequentially aliquoted for DESI-MS in the direction from the cathode to the anode in between 120 to 121 min of the reaction age (see panel c). The mass accuracies of the detected intermediate species are listed in Table S1.

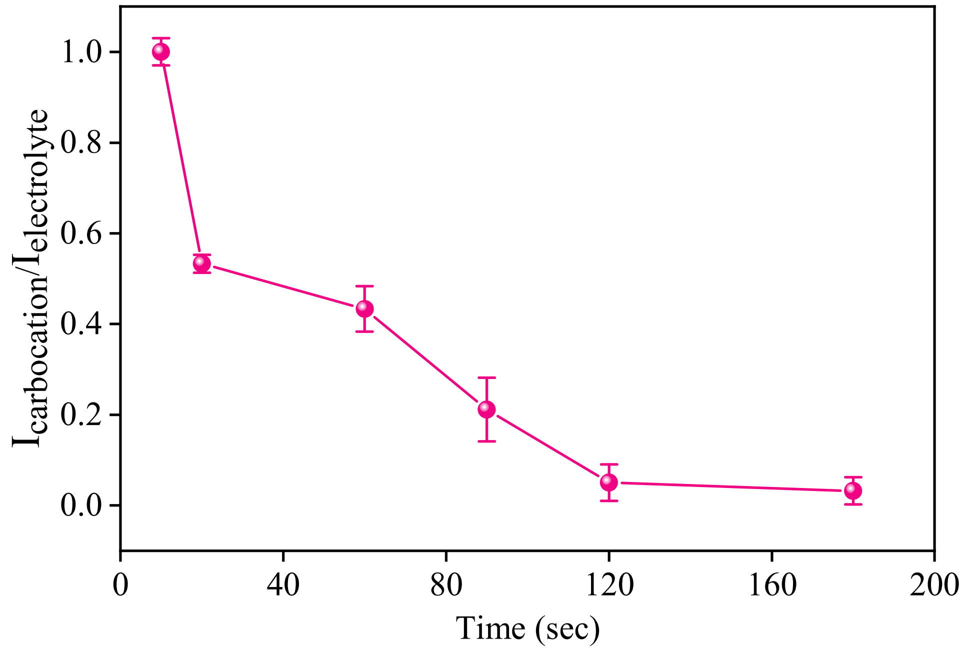

Fig. S12. Time-dependent annihilation of the reactive carbocation intermediate in reaction aliquots withdrawn from the electrolytic cell using Hamilton syringes. A representative electrochemical transformation, such as benzylic C–H amination of ethylbenzene (Fig. 2a), was performed, and at 20 min of the reaction age, six 10 μL aliquots were collected (within 15 s) into separate Hamilton syringes. These aliquots were subsequently analyzed by DESI-MS at different time points corresponding to varying residence times in the syringe. The carbocation ion signal was normalized against the signal of the supporting electrolyte ion (n-Bu_4N^+).

References

1. Kumar, A.; Mondal, S.; Banerjee, S., Efficient Desorption and Capture of Reactive Carbocations from Positively Charged Glass Surface Bombarded with High-Speed Water Microdroplets. *J. Phys. Chem. C* **2023,** *127* (14), 6662-6669.
2. Kumar, A.; Mondal, S.; Sandeep; Venugopalan, P.; Kumar, A.; Banerjee, S., Destabilized Carbocations Caged in Water Microdroplets: Isolation and Real-Time Detection of α-Carbonyl Cation Intermediates. *J. Am. Chem. Soc.* **2022,** *144* (8), 3347-3352.
3. Kumar, A.; Mondal, S.; Mofidfar, M.; Zare, R. N.; Banerjee, S., Capturing Reactive Carbanions by Microdroplets. *J. Am. Chem. Soc.* **2022,** *144* (17), 7573-7577.
4. Kumar, A.; Mondal, S.; Banerjee, S., Aqueous Microdroplets Capture Elusive Carbocations. *J. Am. Chem. Soc.* **2021,** *143* (6), 2459-2463.
5. Hou, Z.-W.; Liu, D.-J.; Xiong, P.; Lai, X.-L.; Song, J.; Xu, H.-C., Site-Selective Electrochemical Benzylic C−H Amination. **2021,** *60* (6), 2943-2947.
6. Meng, L.; Su, J.; Zha, Z.; Zhang, L.; Zhang, Z.; Wang, Z., Direct Electrosynthesis of Ketones from Benzylic Methylenes by Electrooxidative C�H Activation. *Chem. - Euro. J.* **2013,** *19* (18), 5542-5545.
7. Wang, H.; Liang, K.; Xiong, W.; Samanta, S.; Li, W.; Lei, A., Electrochemical oxidation-induced etherification via C(sp3)–H/O–H cross-coupling. *Sci. Adv.* **2020,** *6* (20), eaaz0590.
8. Zhang, Y.; Hou, J.; Yang, H.; Wang, S.; Yuan, K., Electrochemically enhanced deoxygenative cross-coupling of aryl ketones with heteroarenes through in situ generated benzyl carbocations. *Org. Biomol. Chem.* **2023,** *21* (1), 80-84.
9. Shao, X.; Tian, L.; Wang, Y., C–N Coupling of Azoles or Imides with Carbocations Generated by Electrochemical Oxidation. *Euro. J. Org. Chem.* **2019,** *2019* (25), 4089-4094.
10. Yang, Y.-Z.; Wu, Y.-C.; Song, R.-J.; Li, J.-H., Electrochemical dehydrogenative cross-coupling of xanthenes with ketones. *Chem. Commun.* **2020,** *56* (55), 7585-7588.
11. Hong, J. E.; Yoon, J.; Baek, W.; Kim, K.; Kwak, J.-H.; Park, Y., Electrochemical C(sp3)–H Lactonization of 2-Alkylbenzoic Acids toward Phthalides. *Org. Lett.* **2023,** *25* (1), 298-303.
12. Rountree, E. S.; McCarthy, B. D.; Eisenhart, T. T.; Dempsey, J. L., Evaluation of Homogeneous Electrocatalysts by Cyclic Voltammetry. *Inorg. Chem.* **2014,** *53* (19), 9983-10002.
13. Sandford, C.; Edwards, M. A.; Klunder, K. J.; Hickey, D. P.; Li, M.; Barman, K.; Sigman, M. S.; White, H. S.; Minteer, S. D., A synthetic chemist's guide to electroanalytical tools for studying reaction mechanisms. *Chemical Science* **2019,** *10* (26), 6404-6422.
14. Mohamadighader, N.; Zivari-Moshfegh, F.; Nematollahi, D., Electrochemical generation of phenothiazin-5-ium. A sustainable strategy for the synthesis of new bis(phenylsulfonyl)-10H-phenothiazine derivatives. *Sci. Rep.* **2024,** *14* (1), 4276.
15. Stefancu, A.; Aizpurua, J.; Alessandri, I.; Bald, I.; Baumberg, J. J.; Besteiro, L. V.; Christopher, P.; Correa-Duarte, M.; de Nijs, B.; Demetriadou, A.; Frontiera, R. R.; Fukushima, T.; Halas, N. J.; Jain, P. K.; Kim, Z. H.; Kurouski, D.; Lange, H.; Li, J.-F.; Liz-Marzán, L. M.; Lucas, I. T.; Meixner, A. J.; Murakoshi, K.; Nordlander, P.; Peveler, W. J.; Quesada-Cabrera, R.; Ringe, E.; Schatz, G. C.; Schlücker, S.; Schultz, Z. D.; Tan, E. X.; Tian, Z.-Q.; Wang, L.; Weckhuysen, B. M.; Xie, W.; Ling, X. Y.; Zhang, J.; Zhao, Z.; Zhou, R.-Y.; Cortés, E., Impact of Surface Enhanced Raman Spectroscopy in Catalysis. *ACS Nano* **2024,** *18* (43), 29337-29379.
16. Mehara, J.; Roithová, J., Identifying reactive intermediates by mass spectrometry. *Chemical Science* **2020,** *11* (44), 11960-11972.
